# Intrinsically disordered regions restrain genomic targeting of RNA and histone demethylases in mammals and plants

**DOI:** 10.1038/s41588-026-02685-w

**Published:** 2026-07-23

**Authors:** Liudan Jiang, Long Zhao, Jiangbo Wei, Bochen Jiang, Yu Xiao, Fan Yang, Xiaolin Zeng, Chuan He

**Affiliations:** 1 Department of Chemistry, The University of Chicago, Chicago, IL 60637, USA; 2 Howard Hughes Medical Institute, Chicago, IL 60637, USA; 3 Department of Chemistry, Department of Biological Sciences, and Cancer Science Institute, National University of Singapore, Singapore; 4 School of Life Sciences & Biotechnology, Shanghai Jiao Tong University, Shanghai, 200240, China; 5 Sycamore Research Institute of Life Sciences, Shanghai, 201203, China; 6 Department of Chemistry, Fudan University, Shanghai, 200438, China; 7 Department of Biochemistry and Molecular Biology, The University of Chicago, Chicago, IL 60637, USA; 8 Institute for Biophysical Dynamics, The University of Chicago, Chicago, IL 60637, USA; 9 These authors contributed equally

## Abstract

Intrinsically disordered regions (IDRs) are widely recognized as facilitators of chromatin regulation. However, their potential role as regulatory restraints remains poorly understood. Here, we reveal that the C-terminal IDR of mammalian ALKBH5 anchors its main activity to mRNA, limiting chromatin engagement in mouse embryonic stem cells (mESCs). IDR deletion redirects ALKBH5 to chromatin-associated non-coding RNAs, driving widespread chromatin opening and transcriptional activation. This mechanism extends to histone demethylases, where IDR deletion also results in broad removal of repressive histone marks, leading to increased chromatin accessibility and activation of transposable elements (TEs). Building on these insights, we applied IDR deletion to engineer ALKBH5 and its plant homologs. Overexpression of these IDR-deleted ALKBH5 demethylases enhanced *Arabidopsis* root growth and markedly increased rice yield. Our findings uncover a conserved role for IDRs in restraining chromatin access by RNA and histone demethylases and establish a strategy to reprogram chromatin through endogenous plant proteins for crop improvement.

## Introduction

*N*^6^-methyladenosine (m^6^A) is a prevalent RNA modification present on both mRNA and chromatin-associated RNA (caRNA) in higher eukaryotes, regulating gene expression at transcriptional and post-transcriptional levels^1–3^. FTO and ALKBH5, two well-characterized RNA m^6^A demethylases, exhibit distinct substrate preferences and biological functions^2,4–14^. While FTO predominantly demethylates caRNA to modulate chromatin accessibility and transcription during mammalian development^2^, ALKBH5 primarily acts on mRNA across various biological systems^4,5,10–14^. In mouse embryonic stem cells (mESCs), FTO preferentially removes m^6^A from caRNA, with minimal impact on mRNA^2^. In contrast, *Alkbh5* knockout (KO) in mESCs led to a marked increase in m^6^A levels on mRNA, with only a limited effect on caRNA (Extended Data Fig. 1a–c), further confirming the distinct substrate preferences between FTO and ALKBH5.

This divergence extends to plants. Overexpression of mammalian FTO in rice and potatoes has been shown to enhance plant growth and crop yield by demethylating m^6^A on caRNA to affect chromatin states^15^. However, all known plant m^6^A demethylases are homologous to ALKBH5, not FTO, and primarily act on plant mRNA to impact post-transcriptional regulation^16–19^. Consistently, we found in this study that overexpressing either mammalian ALKBH5 or its homologs in plants showed minimum effects on plant growth. Given the regulatory and public acceptance challenges of introducing animal genes into agriculture, deciphering the molecular basis of FTO and ALKBH5 substrate selectivity could inform rational engineering of plant ALKBH5 homologs for improved agricultural traits. Here we reveal a conserved restraining role of IDRs in limiting chromatin engagement by RNA and histone demethylases, establishing a framework to reprogram plant ALKBH5 homologs for chromatin control.

## Results

### ALKBH5 cIDR deletion promotes open chromatin in mESCs

Although both FTO and ALKBH5 contain Fe(II)/α-ketoglutarate-dependent catalytic domains, ALKBH5 uniquely harbors IDRs that are absent in FTO (Fig. 1a). Given that the catalytic domain of ALKBH5 retains m^6^A demethylation activity comparable to the full-length protein^20,21^, IDRs likely modulate other functional properties rather than directly influencing catalytic activity. Since IDRs mediate protein-protein interactions, influence RNA-binding activity of RNA-binding proteins, and regulate subcellular localization of various chromatin-modifying proteins^22^, we hypothesized that ALKBH5 IDRs might contribute to its RNA substrate specificity compared to FTO.

To test this, we ectopically expressed human full-length ALKBH5, FTO, and ALKBH5 variants lacking either the N- or C-terminal IDR in mESCs (Fig. 1b); we used human ALKBH5 and FTO in all ectopic expression experiments in mESCs and plants unless noted otherwise. FTO exhibited a diffuse nuclear localization, whereas full-length ALKBH5 formed distinct nuclear condensates (Fig. 1b). Deletion of the cIDR of ALKBH5 produced a diffuse distribution pattern similar to that of FTO (Fig. 1b), indicating that the cIDR is required for condensate formation. This observation aligns with a recent report showing that ALKBH5 undergoes phase separation through its cIDR in vitro^23^.

To further investigate the function of cIDR, we generated an *Alkbh5*-ΔcIDR mESC line by introducing a stop codon upstream of the cIDR-encoding region at the endogenous *Alkbh5* locus (Extended Data Fig. 1d–f). Surprisingly, deletion of the endogenous cIDR of *Alkbh5* resulted in notable increases in global chromatin accessibility and nascent RNA transcription in mESCs, whereas *Alkbh5* KO had minimal effects (Fig. 1c–d and Extended Data Fig. 1g). These results indicate that the cIDR of mouse ALKBH5 (mALKBH5) likely suppresses its chromatin activation activity.

To understand how cIDR affects RNA-binding specificity, we performed enhanced cross-linking and immunoprecipitation sequencing (eCLIP-seq) on endogenous full-length mALKBH5 and its cIDR-deleted variant (mALKBH5-ΔcIDR) in mESCs. cIDR deletion markedly altered RNA-binding profiles, with only 20% of eCLIP peaks overlapping between the two proteins (Fig. 1e). While both bound near stop codons on mRNA, mALKBH5-ΔcIDR exhibited increased binding at promoter-proximal regions (Fig. 1f), indicating a shift toward cis-regulatory elements (CREs) upon cIDR deletion.

Full-length mALKBH5 preferentially bound m^6^A-modified sites on mRNA, whereas mALKBH5-ΔcIDR exhibited a broader genomic distribution, with enrichment at promoters, enhancers, and transcriptionally active regions (Fig. 1g and Extended Data Fig. 1h–i). mALKBH5-ΔcIDR also displayed increased binding to diverse RNA species, including mRNA, as well as promoter-associated RNA (paRNA), enhancer RNA (eRNA), and repeat RNA—collectively termed chromatin-associated regulatory RNAs (carRNAs)—which are implicated in chromatin regulation (Extended Data Fig. 1j)^2,3^. Moreover, mALKBH5-ΔcIDR-bound regions displayed significantly increased chromatin accessibility and elevated transcription of both mRNA and caRNA (Fig. 1h). Notably, these effects were most pronounced at ΔcIDR-bound regions but also extended up to 100 kb away, suggesting their enhancer-like activity (Fig. 1i).

### cIDR deletion redirects mALKBH5 to chromatin-associated repeat RNAs in mESCs

We next performed m^6^A methylated RNA immunoprecipitation sequencing (MeRIP-seq) to examine how cIDR deletion alters the caRNA m^6^A methylome. Differential m^6^A analysis revealed distinct methylation patterns in *Alkbh5*^−/−^ and *Alkbh5*-ΔcIDR mESCs relative to WT mESCs (Fig. 2a–b). RNAs were categorized into camRNA (chromatin-associated, protein-coding messenger RNA) and carRNAs, including paRNA, eRNA, and repeat RNA. In *Alkbh5* KO mESCs, hypermethylated peaks (sites demethylated by full-length mALKBH5 in WT mESCs) were enriched in camRNA (Fig. 2c) and distributed across exons and introns (Fig. 2d and Extended Data Fig. 2a). In contrast, hypomethylated peaks in *Alkbh5*-ΔcIDR mESCs (sites demethylated by mALKBH5-ΔcIDR) were primarily enriched in carRNAs, particularly repeat RNA (Fig. 2c), with most peaks located in intergenic regions (Fig. 2d). These m^6^A methylation changes inversely correlated with transcriptional output (Fig. 2e–f and Extended Data Fig. 2b). This shift in substrate preference from camRNA to carRNAs following cIDR deletion suggested a potential role for the exon junction complex (EJC), which is a known ALKBH5 interactor involved in mRNA processing^24,25^. We indeed found that hALKBH5 condensates colocalized with the EJC core factor EIF4A3 in mESCs (Extended Data Fig. 2c), and cIDR deletion abolished mALKBH5-EJC interactions (Extended Data Fig. 2d). Consistent with the positioning of the EJC upstream of exon-exon junctions^26,27^, mALKBH5-demethylated m^6^A sites were enriched ~500 nt from splice sites, a pattern lost upon *Alkbh5*-ΔcIDR (Extended Data Fig. 2e). These findings collectively reveal that EJC can mediate ALKBH5 targeting to mRNA in a cIDR-dependent manner.

Most hypomethylated peaks in *Alkbh5*-ΔcIDR mESCs mapped to repeat RNAs and were enriched in evolutionarily young LINE1 elements, which exhibited reduced m^6^A and increased transcription (Fig. 2g–i and Extended Data Fig. 2f–g). Activation of young LINE1s, known regulators of chromatin states^2,3,28–32^, may underlie the widespread chromatin opening and transcriptional upregulation in *Alkbh5*-ΔcIDR mESCs; this parallels our earlier observations in *Fto*^−/−^ mESCs, where mouse FTO (mFTO) demethylates m^6^A on chromatin-associated repeat RNAs, particularly LINE1, to regulate chromatin state and transcription^2^. Similarly, *Alkbh5*-ΔcIDR mESCs exhibited reduced m^6^A levels and elevated transcription at these mFTO-regulated repeat regions (Fig. 2j), suggesting shared m^6^A demethylation activity on repeat RNAs.

We previously showed that the nuclear m^6^A-binding protein YTHDC1 mediates the degradation of methylated repeat RNAs^3^. In *Fto*^−/−^ mESCs, elevated YTHDC1 binding accelerates LINE1 RNA decay, leading to reduced transcription rates^2^. Likewise, *Alkbh5*-ΔcIDR reduced LINE1 RNA decay and YTHDC1 binding (Extended Data Fig. 2h–i). YTHDC1 also recruits histone modifiers that deposit repressive histone marks to suppress local transcription^2,28,33^. Consistently, transcription rate of repeat RNAs, particularly LINE1 RNA, substantially increased upon *Alkbh5*-ΔcIDR, whereas *Alkbh5* KO showed only a modest effect (Fig. 2k–l and Extended Data Fig. 2j). Together, these findings suggest that mALKBH5-ΔcIDR-mediated m^6^A demethylation promotes nascent repeat RNA synthesis and stabilizes these transcripts on chromatin.

Despite functional overlaps between mFTO and mALKBH5-ΔcIDR, their regulatory targets differ. In *Fto*^−/−^ mESCs, hypermethylated peaks are predominantly intronic (Fig. 2d), consistent with mFTO targeting intragenic LINE1 RNA within genes association with differentiation and development^2^. In contrast, mALKBH5-ΔcIDR preferentially regulates intergenic LINE1 (Fig. 2m), suggesting a broader influence on chromatin architecture.

Given these overlapping roles in LINE1 regulation, we asked whether ALKBH5-ΔcIDR could compensate for FTO loss in mESCs. Ectopic expression of human ALKBH5-ΔcIDR partially rescued proliferation and differentiation defects in *Fto*^−/−^ mESCs (Extended Data Fig. 3a–b)^2^, supporting functional similarities between FTO and ALKBH5-ΔcIDR. Conversely, fusing the cIDR of human ALKBH5 to human FTO (FTO-cIDR) conferred ALKBH5-like properties, including nuclear condensate formation, preferential activity on mRNA over caRNA and interaction with EJC components (Extended Data Fig. 3c–f). FTO-cIDR condensates colocalized with the EJC core component EIF4A3 (Extended Data Fig. 3e), and rescued proliferation and differentiation defects in *Alkbh5*^−/−^ mESCs (Extended Data Fig. 3g–j). Together, these findings establish the ALKBH5 cIDR, which appears to exert its effects by promoting nuclear condensate formation, as one of the key determinants of functional divergence between FTO and ALKBH5. The potential impact of condensate properties on RNA substrate specificity or catalytic activity remains to be investigated in the future.

In summary, our findings demonstrate distinct regulatory functions for ALKBH5, FTO and ALKBH5-ΔcIDR within the chromatin-associated fraction of mESCs (Fig. 2n). mALKBH5 primarily demethylates m^6^A on exonic and intronic regions of camRNA, influencing pre-mRNA abundances. mFTO mainly targets intragenic repeat RNAs, whereas mALKBH5-ΔcIDR preferentially engages intergenic repeat RNAs. Both mFTO and mALKBH5-ΔcIDR can notably change chromatin organization and transcription activation.

### m^6^A-hypomethylated LINE1 acts as an enhancer-like element

To probe how mALKBH5-ΔcIDR influences chromatin state in mESCs, we examined m^6^A-hypomethylated regions and found strong overlaps with regions marked by H3K4me3, H3K9me3 and H3K27ac (Fig. 3a). Among them, H3K4me3 and H3K27ac increased globally following *Alkbh5*-ΔcIDR, correlating well with reduced m^6^A levels on caRNA (Fig. 3b–c and Extended Data Fig. 4a–c). LINE1 RNA-targeted genomic sites also displayed increased H3K4me3 and H3K27ac deposition, and enhanced chromatin accessibility upon *Alkbh5*-ΔcIDR (Fig. 3b and Extended Data Fig. 4d). Additionally, chromatin regulators CBP/P300 and YY1, which interact with caRNAs and promote chromatin activation^34,35^, displayed increased occupancy at regions with elevated H3K4me3 and H3K27ac levels following *Alkbh5-*ΔcIDR (Fig. 3d–e).

Notably, m^6^A-marked LINE1 elements exhibited increased H3K27ac following *Alkbh5*-ΔcIDR, a feature of active enhancers (Fig. 3f). Given that LINE1 can regulate distal gene transcription through enhancer-promoter looping^32^, we examined whether m^6^A-hypomethylated LINE1 RNA acts as an enhancer via such looping interactions. We integrated Pol II ChIA-PET data, which map promoter-enhancer loops linked to active transcription. We specifically analyzed chromatin loops where one of the two anchor sites overlapped with m^6^A-hypomethylated LINE1 regions following *Alkbh5*-ΔcIDR, defining these as “m^6^A-hypomethylated LINE1-related loops”. Notably, 85% of these anchors overlapped with enhancer regions (Fig. 3g). To examine chromatin and transcriptional changes at these loops, we overlaid ATAC-seq, H3K27ac and H3K4me3 CUT&Tag, and transcriptome data. Loop-associated regions exhibited increased chromatin accessibility, elevated H3K4me3 intensity, and enhanced transcription of protein-coding genes compared to non-interacting regions (Fig. 3h–i). Furthermore, these activated genes were enriched in transcription and chromatin regulation processes (Fig. 3j), potentially amplifying the downstream regulatory effects of hypomethylated LINE1. Consistently, time-course nascent RNA profiling revealed earlier and stronger induction of transcriptional activators upon *Alkbh5*-ΔcIDR (Extended Data Fig. 4e).

To establish causality, we targeted representative loop-related LINE1 loci in WT mESCs using dCas13d-ALKBH5-ΔcIDR and proper guide RNA (Extended Data Fig. 4f). This targeted recruitment of ALKBH5-ΔcIDR reduced local m^6^A and activated distal looped genes, recapitulating the effects observed in *Alkbh5*-ΔcIDR mESCs, whereas its catalytically inactive mutant had no such effect (Extended Data Fig. 4g–n). Globally, genes activated in *Alkbh5*-ΔcIDR mESCs were enriched in translation, transcription, and cell cycle regulation (Extended Data Fig. 4o). Altogether, these findings support a feedback loop initiated by local RNA demethylation at LINE1 RNA, sustaining chromatin activation and influencing cellular growth and development.

Therefore, cIDR deletion releases ALKBH5 to demethylate LINE1 RNA (Fig. 3k). These m^6^A-hypomethylated LINE1 RNA, evading YTHDC1-mediated degradation, promote chromatin opening, enhance H3K27ac and H3K4me3 deposition, and recruit transcriptional activators P300 and YY1. These loci function like enhancers, regulating distal gene transcription through enhancer-promoter looping.

### IDRs restrain genomic targeting of histone demethylases

The widespread chromatin and transcriptional activation observed in *Alkbh5*-ΔcIDR mESCs highlights the critical role of IDRs in preventing demethylation at TEs and maintaining their silencing. TEs constitute nearly half of mammalian genomes and can function as CREs, requiring tight transcriptional control for genome stability^36^. A recent study showed that the IDR of TET3 suppresses its dioxygenase activity, and its loss leads to retrotransposons derepression^37^. While DNA methylation provides a stable and heritable mechanism for silencing, histone modifications represent a more dynamic and adaptable form of chromatin regulation. We hypothesized that IDRs within gene-activating histone demethylases might similarly prevent aberrant removal of repressive histone marks and maintain silenced chromatin state at TEs.

Strikingly, all lysine demethylases (KDMs) contain IDRs outside their catalytic domains (Extended Data Fig. 5a)^38^. We focused on human KDM4A/KDM4B (H3K9me3 demethylases) and KDM6A/KDM6B (H3K27me3 demethylases) due to their well-characterized gene-activating functions and their ability to form nuclear condensates via IDRs (Extended Data Fig. 5b)^38–41^. Transient expression of HA-tagged human full-length and ΔIDR variants in HEK293T cells showed that both forms of KDM4A/KDM4B and KDM6A/KDM6B effectively reduced global H3K9me3 and H3K27me3 levels, respectively, with ΔIDR variants causing more extensive demethylation (Extended Data Fig. 5c–d).

To systematically compare these effects, we generated mESC lines ectopically expressing HA-tagged human full-length and ΔIDR variants (Extended Data Fig. 5c). Deletion of IDRs led to widespread reductions in H3K9me3 for KDM4A/KDM4B and H3K27me3 for KDM6A/KDM6B (Extended Data Fig. 5e). Notably, these histone demethylations were predominantly observed at TEs (Fig. 4a). To study how IDRs regulate chromatin engagement, we performed HA-tag CUT&RUN assays to compare the chromatin-binding profiles of ΔIDR variants and their full-length controls. Full-length and ΔIDR variants showed distinct chromatin-binding profiles for both KDM4A/KDM4B and KDM6A/KDM6B (Fig. 4b and Extended Data Fig. 5f). Of note, ΔIDR variants of KDM4A and KDM6B showed substantially increased chromatin binding in mESCs compared to their respective full-length proteins, correlating with reduced H3K9me3 (KDM4A-ΔIDR) and H3K27me3 (KDM6B-ΔIDR) levels (Fig. 4c–d). Importantly, the increased binding of ΔIDR variants for both KDM4A/KDM4B and KDM6A/KDM6B were mainly localized to TEs (Fig. 4e). Moreover, these aberrant chromatin interactions profoundly altered chromatin states (Extended Data Fig. 5g). Globally, KDM4B/KDM6A/KDM6B-ΔIDR expression led to significantly elevated chromatin accessibility compared to their respective full-length variants, whereas KDM4A variants showed no substantial global change (Fig. 4f). Given the dual activity of KDM4A/4B in demethylating both H3K9me2/3 (repressive) and H3K36me2/3 (activating), we specifically examined H3K9me3 dynamics. A negative correlation was observed between changes in H3K9me3 signals and chromatin accessibility upon KDM4A/4B-ΔIDR expression (Fig. 4g). Furthermore, the increased chromatin accessibility induced by ΔIDR variants for both KDM4A/4B and KDM6A/6B led to the activation of TEs (Fig. 4h).

H3K9me3 is essential for silencing TEs in mESCs, particularly at long terminal repeats (LTRs) and LINE1 elements^42,43^. Consistent with this, the broader removal of H3K9me3 induced by KDM4A/4B-ΔIDR was concentrated at LTRs and LINE1 elements (Extended Data Fig. 5h). IDRs apparently ensure proper genomic targeting of gene-activating histone demethylases by their proper recruitment to gene activation loci while limiting broad chromatin engagement to prevent aberrant demethylation at TEs (Fig. 4i). This restriction preserves TEs silencing and safeguards genomic stability by preventing their activation as putative CREs that could drive aberrant transcriptional activation.

### Engineering ALKBH5 and its plant homologs for carRNA m^6^A demethylation

Our comparative analysis in mESCs demonstrates that IDRs restrain the chromatin engagement of ALKBH5 and gene-activating histone demethylases. This prompted us to ask whether plant ALKBH5 homologs could be engineered to acquire animal FTO-like chromatin regulatory activity to enhance plant growth^15^.

We developed a workflow to functionally assess engineered ALKBH5 homologs (Fig. 5a). These constructs were designed with nuclear localization signals (NLS) to ensure nuclear entry—a prerequisite for chromatin targeting, and with IDR deletions to enable broader m^6^A demethylation. mESCs were used in an initial screen, where LINE1 RNAs serve as major substrates of FTO and ALKBH5-ΔcIDR (Fig. 2)^2^ to provide a robust readout of potential chromatin regulatory activity. We selected constructs that significantly elevated LINE1 RNA levels for further testing in plants. To rapidly assess their effects in plants, we transiently expressed engineered demethylases in tobacco (*Nicotiana benthamiana*) leaves, a system with high transformation efficiency and robust protein expression^44^. We performed DNase I-treated TUNEL assays to determine global chromatin state changes. Constructs that promoted chromatin opening were then stably transformed into *Arabidopsis* for growth evaluation. Since overexpression of mammalian FTO greatly enhanced root growth in rice^15^, and *Arabidopsis* grows faster, we used root growth in *Arabidopsis* as a functional readout to assess the engineered demethylases in promoting plant growth.

We validated our workflow using human FTO and ALKBH5 as benchmarks. Ectopic expression of human FTO or IDR-deleted ALKBH5 variants (ALKBH5_Δ30–81_, ALKBH5_Δ298–394_, and ALKBH5_Δ30–81 and 298–394_) in mESCs elevated whole-cell LINE1 RNA levels and reduced m^6^A on chromatin-associated LINE1 RNAs (Fig. 5b–c and Extended Data Fig. 6a–c). In contrast, full-length ALKBH5 and catalytically inactive mutants (H204A) had no effect (Fig. 5c and Extended Data Fig. 6d). In tobacco leaves, expression of human FTO or IDR-deleted ALKBH5 variants enhanced chromatin accessibility (Fig. 5d–e and Extended Data Fig. 6e). Consistent with observations in *FTO*-transgenic rice^15^, 10-day-old *FTO*-transgenic *Arabidopsis* displayed increased primary root length (Fig. 5f and Extended Data Fig. 6f). Similarly, transgenic expression of ALKBH5_Δ298–394_ and ALKBH5_Δ30–81 and 298–394_ promoted root elongation, whereas full-length or catalytically inactive mutants did not (Fig. 5g–i and Extended Data Fig. 6f–m). Notably, cIDR deletion appears to be critical for promoting root growth, as ALKBH5_Δ30–81_ had only a limited effect (Extended Data Fig. 6h–i).

### IDR-deleted ALKBH5 reprograms chromatin in *Arabidopsis*

We next investigated how IDR deletion promotes root growth in transgenic *Arabidopsis*. As in mESCs, we focused on human *ALKBH5*_Δ298–394_-transgenic *Arabidopsis* (hereafter “*ALKBH5*-ΔcIDR”), with full-length *ALKBH5*-transgenic and catalytically inactive mutant lines serving as controls. Compared to full-length ALKBH5, ALKBH5-ΔcIDR exhibited dramatically enhanced chromatin binding in *Arabidopsis* (24,590 versus 2,057 binding sites) (Fig. 6a). CUT&Tag profiling revealed global increases in H3K4me3 and H3K27ac levels in *ALKBH5*-ΔcIDR-*Arabidopsis* compared to full-length *ALKBH5*-*Arabidopsis*, along with enhanced chromatin accessibility (Fig. 6b and Extended Data Fig. 7a–c). Elevated H3K4me3 and H3K27ac signals correlated with increased chromatin binding of ALKBH5-ΔcIDR (Fig. 6c and Extended Data Fig. 7d), revealing that broader chromatin targeting promotes active chromatin state.

We then performed caRNA MeRIP-seq in *ALKBH5*- and *ALKBH5*-ΔcIDR-*Arabidopsis*, along with their respective inactive mutant lines. Compared to full-length *ALKBH5*-*Arabidopsis*, *ALKBH5*-ΔcIDR-*Arabidopsis* exhibited greater m^6^A demethylation on caRNA, accompanied by increased transcription of m^6^A-marked transcripts and elevated H3K27ac at m^6^A-hypomethylated regions (Fig. 6d and Extended Data Fig. 7e). Notably, small nucleolar RNA (snoRNAs) and small nuclear RNA (snRNAs) displayed reduced m^6^A levels alongside elevated transcription in *ALKBH5*-ΔcIDR-*Arabidopsis* (Fig. 6e). Given the limited TE content in *Arabidopsis* and potential cis-regulatory roles of snoRNAs and snRNAs on chromatin state^45,46^, these m^6^A-hypomethylated non-coding RNAs may reinforce local chromatin activation.

Although both transgenic lines showed elevated transcript levels, ALKBH5-ΔcIDR-mediated m^6^A demethylation resulted in distinct transcriptional profiles (Fig. 6f). Genes activated in *ALKBH5*-ΔcIDR-*Arabidopsis* were enriched in pathways related to photosynthesis, plant development, and transcriptional and translational regulation (Fig. 6g). The enrichment of transcriptional and translational regulators suggests a potential amplifying effect, aligning with our findings in mESCs.

To examine chromatin effects of caRNA hypomethylation, we analyzed previously identified chromatin interaction sites of top-ranked m^6^A-hypomethylated RNAs (e.g., At*3g56825* (U2.4), At*3g56705* (U2.6), At*5g61455* (U2.7))^46^. Over half of these sites were marked by H3K4me3 and H3K27ac, with both marks showing increased levels in *ALKBH5*-ΔcIDR-*Arabidopsis* compared to *ALKBH5*-*Arabidopsis* (Fig. 6h and Extended Data Fig. 7f–g).

Collectively, we show that ALKBH5-ΔcIDR-mediated m^6^A demethylation elevates a subset of caRNAs. These caRNAs may function as CRE-like regulators, modulating chromatin states and activating transcriptional programs associated with enhanced growth in *Arabidopsis*.

### Engineering *Arabidopsis* and rice ALKBH5 orthologs

We next explored whether comparable growth enhancement could be achieved by manipulating plant ALKBH5 orthologs. *Arabidopsis* encodes two m^6^A demethylases, ALKBH9B and ALKBH10B, which share conserved catalytic domains with mammalian ALKBH5^16,19^. BLAST analysis identified two putative ALKBH5 orthologs in rice: LOC_Os06g04660 (hereafter “Os9B”) and LOC_Os10g02760 (hereafter “Os10B”) (Extended Data Fig. 8a). Biochemistry assays confirmed that both Os9B and Os10B possess RNA m^6^A demethylation activity (Extended Data Fig. 8b), consistent with recent reports on Os9B^17,18^. Similar to ALKBH5, these orthologs contain IDRs outside their catalytic domains (Extended Data Fig. 8c).

Leveraging insights from human ALKBH5, we engineered *Arabidopsis* ALKBH9B, rice Os9B, and rice Os10B constructs with N-terminal NLS and IDR deletions to assess chromatin-modulatory activity (Extended Data Fig. 8d). In mESCs, expression of truncated variants of ALKBH9B (ALKBH9B_Δ76–102_, ALKBH9B_Δ145–183_ and ALKBH9B_Δ432–507_), Os9B (Os9B_Δ428–616_ and Os9B_Δ60–170 and 428–616_), and Os10B (Os10B_Δ491–595_ and Os10B_Δ2–30, 99–126 and 491–595_) markedly elevated whole-cell LINE1 RNA levels in m^6^A-dependent manners (Fig. 7a–b and Extended Data Fig. 8e–h). Among the two *Arabidopsis* m^6^A demethylases, we prioritized ALKBH9B due to its higher activity to upregulate LINE1 RNA abundance in mESCs (Fig. 7a, Extended Data Fig. 8i). Although the native *Arabidopsis* ALKBH9B is cytoplasmic, NLS addition redirected it to the nucleus in mESCs and *Arabidopsis* protoplast (Extended Data Fig. 8j). Subsequent cIDR deletion shifted its nuclear distribution from condensates to a diffuse pattern (Extended Data Fig. 8j), confirming the effectiveness of our engineering strategy. Furthermore, expression of these IDR-deleted ALKBH9B, Os9B, and Os10B variants in tobacco leaves induced chromatin opening (Fig. 7c–d and Extended Data Fig. 8k). We next generated stable transgenic *Arabidopsis* lines expressing these variants (Extended Data Fig. 9a). IDR-deleted variants, ALKBH9B_Δ432–507_, Os9B_Δ428–616_, Os9B_Δ60–170 and 428–616_, Os10B_Δ491–595_ and Os10B_Δ2–30, 99–126 and 491–595_, markedly promoted primary root growth in *Arabidopsis* (Fig. 7e–g and Extended Data Fig. 9b). Again, full-length and catalytically inactive variants showed limited effects across these assessed phenotypes (Extended Data Fig. 8f–h, 8k, 9b).

We next performed MeRIP-qPCR on top-ranked m^6^A-hypomethylated loci identified in *ALKBH5*-ΔcIDR-*Arabidopsis*. We focused on transgenic *Arabidopsis* lines expressing ALKBH9B_Δ432–507_, Os9B_Δ428–616_, and Os10B_Δ491–595_. These cIDR-deleted lines exhibited markedly reduced m^6^A levels on caRNAs at these loci compared to their full-length counterparts (Extended Data Fig. 9c). These reductions were accompanied by elevated transcript levels and increased H3K27ac and H3K4me3 signals, indicating more active chromatin states upon cIDR deletion (Extended Data Fig. 9d–e).

### Transgenic expression of IDR-deleted ALKBH5 increases rice yield

Given the success of *FTO*-transgenic approach in increasing rice yield^15^, we next explored whether IDR-deleted ALKBH5 could deliver similar agronomic benefits. Using human ALKBH5 as a benchmark, we generated transgenic rice lines in the Zhonghua11 (ZH11) background, expressing full-length or IDR-deleted ALKBH5 (ALKBH5_Δ298–394_ and ALKBH5_Δ30–81 and 298–394_), alongside catalytically inactive mutants as controls (Extended Data Fig. 10a). Consistent with observations in *Arabidopsis*, *ALKBH5*_*Δ298–394*_-transgenic rice seedlings exhibited significantly longer roots compared to WT (ZH11) and *ALKBH5*_*Δ298–394*_^*mut*^-transgenic rice (Fig. 7h and Extended Data Fig. 10b). Moreover, *ALKBH5*_Δ298–394_- and *ALKBH5*_Δ30–81 and 298–394_-transgenic rice displayed higher photosynthesis rates at the filling stage under both greenhouse and field conditions (Extended Data Fig. 10c–e), along with increased tiller numbers compared to WT and their respective inactive mutant lines (Fig. 7i and Extended Data Fig. 10f–g). These improvements culminated in significantly higher grain yields (45–55% increases in greenhouse, 25–30% increases in the field) and biomass (25–40% increases in greenhouse, 25–30% increases in the field) (Fig. 7j–k and Extended Data Fig. 10h–k). In contrast, full-length ALKBH5 showed no significant effects on these agronomic traits.

## Discussion

Our study uncovers an underexplored role of IDRs as regulatory restraints on gene-activating demethylases, limiting chromatin engagement and preserving genomic stability. This function is a key determinant of the distinct substrate preferences and regulatory activities of FTO and ALKBH5 in mammalian and plants. In mESCs, cIDR deletion redirects ALKBH5 from mRNA to chromatin-associated intergenic LINE1 RNAs, triggering enhancer-like activity and widespread chromatin remodeling. Similarly, removing IDRs from gene-activating histone demethylases disrupts chromatin targeting and activates TEs transcription. Very recent studies also implicated IDRs in TET family DNA demethylases in preventing uncontrolled DNA demethylation^37,47^. These profound consequences of IDR deletion raise the possibility that the IDR-mediated regulatory restraint may be a shared mechanism across diverse chromatin-associated proteins to ensure controlled chromatin modulation. For example, in transcription factors, IDRs are well known to promote the formation of transcriptional condensates for gene activation^48^, and to direct chromatin binding via multivalent weak interactions with DNA^48,49^. While this facilitation-based model represents a major way in which condensates influence genomic features, our findings add another perspective that IDR-encoded constraints may also restrict inappropriate condensate mixing and help prevent unwanted chromatin activation. Further studies are required to elucidate how different IDRs balance molecular flexibility and regulatory restraint in the fine-tuning of chromatin architecture.

The chromatin regulation insights learned on ALKBH5 and FTO in mESCs extend to plant systems. While human *FTO*-transgenic rice has shown the promise of manipulating m^6^A demethylation to improve crop yield and biomass^15^, its reliance on mammalian genes may limit broader applicability and cause compliance concerns. To address this limitation, we leveraged mechanistic insights learned from ALKBH5 regulation in mESCs and engineered both human ALKBH5 and plant ALKBH5 homologs to regulate chromatin state and transcription in plants. Specifically, our findings reveal that IDR-deleted ALKBH5 induces widespread m^6^A demethylation on caRNAs in mESCs and *Arabidopsis*, which promotes chromatin opening and transcription. We show that these caRNAs can act as regulatory elements akin to CREs in modulating chromatin dynamics.

Importantly, these insights guided us towards an IDR deletion strategy to engineer plant ALKBH5 homologs for achieving chromatin activation and plant growth promotion. By constructing IDR-deleted ALKBH5 homologs in *Arabidopsis* and rice, we validated the adaptability of this approach across plant species. These engineered ALKBH5 homologs promote chromatin accessibility and *Arabidopsis* growth. We further confirmed significant photosynthesis increase and yield improvement in rice with transgenic expression of IDR-deleted human ALKBH5, further supporting the utility of IDR deletion strategies for crop enhancement. Given that all known plant m^6^A demethylases are ALKBH5 homologs^16–19^, this approach opens an avenue for reprogramming chromatin dynamics to boost agricultural productivity using engineered plant proteins.

In summary, our study not only provides mechanistic insights into IDRs that play regulatory restrain roles in RNA and histone demethylases to preserve chromatin state but also outlines a practical framework for engineering ALKBH5 family demethylases for agricultural applications. We demonstrated the potential of the IDR-deleted ALKBH5 and its plant homologs to enhance plant growth and crop yield. These findings have implications for future chromatin reprogramming in both animal and plant systems using engineered demethylases.

## Methods

This research complies with study protocols approved by the University of Chicago Institutional Biosafety Committee under protocol number IBC1716 and IBC0951.

### Cell culture

E14TG2a mES cells (ATCC, CRL-1821) were cultured in DMEM (Gibco, 11995065) supplemented with 15% FBS (Gibco, 16141079), 1% EmbryoMax nucleosides (Sigma, ES-008), 1% GlutaMAX supplement (Gibco, 35050061), 1% MEM non-essential amino acids solution (Gibco, 11140050), 1% Penicillin-Streptomycin (Gibco, 15140122), 0.1 mM 2-Mercaptoethanol (Gibco, 21985023), 1000 U/ml LIF (Sigma, ESG1107), 1 μM PD0325901 (Stemcell, 72184) and 3 μM CHIR99021 (Stemcell, 100–1042) at 37 °C in a humidified atmosphere with 5% CO_2_. HEK293T cells (ATCC, CRL-1573) were maintained in DMEM (Gibco, 11995065) supplemented with 10% FBS (Gibco, 26140079) and 1% Penicillin-Streptomycin (Gibco, 15140122) at 37 °C with 5% CO_2_.

### Plasmid construction

All constructs expressed in mESCs were generated using the PPB-CAG-IRES-Pac backbone, which includes puromycin resistance, as well as HA and Flag tags. For engineering ALKBH5 homologs, a triple SV40 NLS sequence was incorporated downstream of the Flag-HA sequence in the backbone. Coding sequences for human FTO and ALKBH5 were amplified from HEK293T cDNA. *Arabidopsis* ALKBH9B coding sequences were synthesized by GenScript, while rice Os9B and Os10B coding sequences were amplified from GenScript ORF clones. Coding sequences for KDM4A/4B/6A/6B were amplified from plasmids #101051, #24181, #24168, #24167 (Addgene) respectively. These fragments were inserted into BglII/XhoI-linearized backbones using NEBBuilder HiFi DNA assembly master mix (NEB, E2621L). Guide RNAs targeting LINE1 loci for dCas13d were clone into AgeI/EcoRI-linearized Tet-pLKO-neo (Addgene #21916) through Gibson assembly.

Constructs intended for plant expression were generated using a *35S::Flag-NLS* backbone. Fragments were amplified from corresponding plasmids tested in mESCs and assembled into BglII/BamHI-linearized plant backbones via Gibson assembly.

All plasmids were verified through either whole-plasmid or Sanger sequencing. A complete list of oligonucleotides used for cloning is provided in Supplementary Table 1.

### Construction of stable cell lines

For ectopic expression cell lines, individual piggyBac plasmids were co-transfected with the pCMV-PBase plasmid encoding a transposase gene at a 1:1 ratio into mESCs using Lipofectamine 3000 (Thermo Scientific, L3000015) according to the manufacturer’s protocol. 48h post-transfection, mESCs were selected with 2 μg/ml puromycin (Gibco, A1113803). After 5–7 days of continuous selection, surviving cells were pooled for subsequent experiments.

For *Alkbh5* KO in mESCs, sgRNAs were clones into the pSpCas9(BB)-2A-Puro plasmid (Addgene #62988) and transfected into mESCs using Lipofectamine 3000 (Thermo Scientific, L3000015), following the manufacturer’s instructions. Following 48h of selection with 2 μg/ml puromycin (Gibco, A1113803), single-cell clones were isolated and verified by western blotting and Sanger sequencing.

For the generation of HA-tagged stop codon knock-in in mESCs, CRISPR-Cas9 mediated homology-directed repair was performed as previously described with modifications. Briefly, crRNA, tracrRNA, ssDNA donor, Alt-R Cas9 enzyme and Alt-R HDR enhancer V2 were obtained from IDT. Active ribonucleoprotein complexes were assembled and transfected into 6 × 10^4^ mESCs along with 0.5 μl of 100 μM donor using the P3 Primary Cell 4D-Nucleofector X Kit S (Lonza, V4XP-3032) according to the manufacturer’s protocol. 48h post-nucleofection, single-cell clones were isolated and verified by western blotting and Sanger sequencing.

For the generation of mESCs stably expressing dCas13d-ALKBH5-ΔcIDR or dCas13d-ALKBH5-ΔcIDR^mut^, piggyBac plasmids encoding the respective fusion proteins were co-transfected with the pCMV-PBase plasmid (1:1 molar ratio) into mESCs using Lipofectamine 3000 (Thermo Scientific, L3000015) following the manufacturer’s protocol. mESCs were selected with 2 μg/ml puromycin (Gibco, A1113803) for 7 days. Surviving cells were transduced with lentivirus carrying individual guide RNA targeting LINE1 loci. At 24h post-transduction, mESCs were selected with 500 μg/ml geneticin (Gibco, 10131035) for 2 weeks to establish stable lines.

Details of sgRNAs, ssDNA donors, and genotyping primers are provided in Supplementary Table 1. Newly generated cell lines used in this study are available upon request from the corresponding author.

### EB differentiation

mESCs were dissociated using TrypLE (Thermo Scientific, 12605036) and cultured in hanging drops on the lid of a Petri dish for 2 days in mESC medium lacking LIF and 2i. The resulting EBs were collected and transferred to ultra-low attachment plates (Corning, 3471) for an additional 5–7 days. Afterward, EBs were harvested for gene expression analysis.

### Cell proliferation assay

mESCs were dissociated using TrypLE (Thermo Scientific, 12605036) and seeded into 96-well plates at a density of 2,500 or 5,000 cells per well. Proliferation assays were conducted using the CellTiter 96 AQueous One Solution Reagent (Promega, G3580) according to the manufacturer’s instructions. Absorbance was measured at 24-, 48-, 72-, and 96-hours post-plating. All absorbance values were normalized to those measured 24 hours post-plating.

### Immunofluorescence staining

mESCs were seeded onto Matrigel-coated 8-well chambers (Ibidi, 80807), fixed with 4% PFA (Thermo Scientific, I28800) for 15 min, and permeabilized with 0.3% Triton X-100 (Sigma, X100) in PBS for 15 min. Following permeabilization, cells were blocked in IF buffer (3% BSA in PBST) for 1 h. Cells were then incubated overnight at 4 °C with primary antibody diluted in IF buffer. The next day, cells were washed three times with PBST, and incubated with secondary antibody diluted in IF buffer for 1h at room temperature. After incubation, cells were washed three times with PBST, and nuclei were counterstained with DAPI (Thermo Scientific, 62248). Fluorescent images were acquired using Leica SP8 confocal microscope.

### Quantitative analysis of m^6^A level using LC-MS/MS

50 ng of non-ribosomal RNA were digested with 1 μl of Nuclease P1 (NEB) in 20 μl of 1× Nuclease P1 buffer (NEB, M0660S) at 37 °C for 2 h. Following Nuclease P1 digestion, 1 μl of FastAP (Thermo Scientific, EF0651) and 2.5 μl of FastAP buffer (Thermo Scientific, EF0651) were added, and the mixture was incubated at 37°C for an additional 4 hours. The resulting solution was filtered through a 0.22 μm syringe filter and diluted to a final volume of 60 μl. Subsequently, 10 μl of the prepared solution was injected into an LC-MS/MS system for analysis. Nucleosides were separated using reverse-phase ultra-performance liquid chromatography on a C18 column and detected via mass spectrometry using an Agilent 6410 QQQ triple-quadruple LC-MS system in positive electrospray ionization mode. The nucleosides were quantified based on the ion mass transitions of 282 to 150 (m^6^A), and 268 to 136 (A). Quantification was performed by comparing the sample results to a standard curve generated using pure nucleoside standards analyzed within the same batch. The ratio of m^6^A to A was calculated from the determined concentrations.

### Cell Fractionation

mESCs were fractionated as previously described^50^ with modifications. Briefly, cells were collected and washed once with PBS containing 1mM EDTA. Then cells were transferred to a 2 ml tube and centrifuged at 500 × g for 3 min at 4°C to collect the cell pellet. The pellet was resuspended in 200 μl of ice-cold lysis buffer (10 mM Tris-HCl, pH = 7.5, 0.05% NP40, 150 mM NaCl, 1× protease inhibitor, 0.2 U/μl SUPERase•In RNase inhibitor) and incubated on ice for 5 min. Next, 2.5 volumes of ice-cold sucrose cushion (24% RNase-free sucrose in lysis buffer) were gently layered beneath the lysis mixture and centrifuged at 5,000 × g for 10 minutes at 4 °C. The resulting supernatant was collected as the cytoplasmic fraction. The nuclei pellet was washed twice with 1 ml of ice-cold PBS containing 1mM EDTA. The nuclei pellet was then resuspended in 200 μl of ice-cold glycerol buffer (20 mM Tris-HCl, pH = 7.9, 75 mM NaCl, 0.5 mM EDTA, 0.5 mM DTT, 50% glycerol, 1× protease inhibitor, 0.2 U/μl SUPERase•In RNase inhibitor) with gentle flicking. Two volumes of cold nuclei lysis buffer (10 mM HEPES, pH = 7.6, 1 mM DTT, 7.5 mM MgCl_2_, 0.2 mM EDTA, 0.3 M NaCl, 1 M UREA, 1% NP-40, 1× protease inhibitor, 0.2 U/μl SUPERase•In RNase inhibitor) was added, followed by vigorously vortexing for 5 seconds twice. The nuclei lysate was incubated on ice for 2 min and centrifuged at 15,000 g for 2 min at 4 °C. The supernatant was collected as the soluble nuclear fraction (nucleoplasm). The remaining pellet was gently rinsed twice with cold PBS containing 1 mM EDTA, and was collected as the chromosome-associated fraction.

### RT-qPCR and MeRIP-qPCR

Whole-cell or specific fractions of mESCs were lysed in TRIzol Reagent (Invitrogen, 15596018), and RNA was extracted using a combination of chloroform extraction and the RNA clean & Concentrate kit (Zymo research, R1013), following the manufacturer’s instructions. To eliminate residual DNA contamination, RNA was treated on-column with DNase I. cDNA synthesis was carried out using the PrimeScript RT Master Mix (Takara, RR036B). Quantitative real-time PCR was performed using FastStart Essential DNA Green Master (Roche, 6924204001) on a LightCycler 96 system (Roche). m^6^A-IP of non-ribosomal RNA from the chromatin-associated fraction of mESCs or *Arabidopsis* was performed using the EpiMark N6-Methyladenosine Enrichment Kit (NEB) following the manufacturer’s instructions. m^6^A and non-m^6^A spike-ins from this kit were used as the normalization controls for m^6^A level analysis in MeRIP-RT-qPCR. Relative gene expression changes were calculated using the ΔΔC_t_ method. Primer sequences used for RT-qPCR are provided in Supplementary Table 1.

### Western blotting

Cells were washed with PBS and lysed with 1×NuPAGE LDS Sample Buffer (Invitrogen, NP0007) containing 1mM DTT to generate whole cell lysate. For co-immunoprecipitation experiments, proteins were eluted from beads by boiling in 2×NuPAGE LDS Sample Buffer (Invitrogen, NP0007) containing 1mM DTT at 95°C for 10 min. For plant samples, tissues were frozen in liquid nitrogen, disrupted using Tissuelyser II (Qiagen). Then the resulting powders were mixed with 2×NuPAGE LDS Sample Buffer (Invitrogen, NP0007) containing 1mM DTT and heated at 95°C for 15 min.

Protein samples were separated on NuPAGE 4 to 12% Bis-Tris gels or NuPAGE 3–8% Tris-Acetate Protein Gels (Invitrogen), and subsequently transferred onto 0.45 μm PVDF membranes using semidry or wet electroblotting (Bio-Rad). Membranes were blocked in TBST containing 5% milk powder at room temperature (RT) for 1h. Subsequently, membranes were incubated overnight at 4°C with primary antibody diluted in TBST containing 3% BSA. The next day, membranes were washed three times with TBST, and incubated with secondary antibody diluted in TBST containing 3% milk powder for 1h at RT. Following incubation, membranes were washed three times with TBST, and imaged using iBright CL1500 Imaging System (Invitrogen).

### Co-immunoprecipitation

Cells were harvested, and nuclei were isolated following the cell fractionation protocol. The isolated nuclei were resuspended in Nuclei Lysis Buffer C (20 mM Tris-Cl pH 7.5, 10% glycerol, 420 mM NaCl, 4 mM MgCl₂, 0.2 mM EDTA, 0.5 mM DTT, and 1× protease inhibitor) and incubated on ice for 30 minutes. The soluble nuclear extract (nuclear extract A) was obtained by centrifugation at 15,000 g for 15 minutes at 4 °C. The remaining insoluble chromatin fraction was resuspended in Buffer A (10 mM Tris-Cl, pH 7.5, 150 mM NaCl, 1.5 mM MgCl₂, 0.5 mM DTT, and 1× protease inhibitor) at two volumes of Nuclei Lysis Buffer C, followed by DNase I treatment. After a 30-minute incubation at 37 °C, the soluble chromatin fraction (nuclear extract B) was collected via centrifugation at 15,000 g for 15 minutes at 4 °C. Nuclear extracts A and B were combined and incubated with the appropriate antibody or IgG control pre-bound to Protein A/G beads at room temperature for 2 hours. The beads were washed four times with wash buffer, prepared by mixing Buffer A and Buffer C in a 2:1 ratio. Finally, 2×NuPAGE LDS Sample Buffer (Invitrogen) containing 1mM DTT was added directly to the beads, and the samples were boiled at 95 °C for 10 minutes before further analysis.

### Nascent RNA synthesis assay

mESCs were seeded onto Matrigel-coated 8-well chambers (Ibidi) one day prior to EU labeling. The nascent RNA synthesis assay was conducted the following day using the Click-iT RNA Alexa Fluor 594 Imaging Kit (Invitrogen, C10330) according to the manufacturer’s instructions. Cell nuclei were counterstained with DAPI (Thermo Scientific, 62248). Fluorescent images were captured using a Leica SP8 confocal microscope, and the intensity of the EU signal was quantified using Fiji software.

### DNase I-treated TUNEL assays

For mESCs, the DNase I-treated TUNEL assay was conducted using the DeadEnd Fluorometric TUNEL System (Promega, G3250) according to the manufacturer’s instructions. Prior to rTdT labeling, cells were treated with 1 U/μl DNase I (Thermo Scientific) at 37 °C for 5min. Flow cytometry was performed using a BD Fortessa (BD), and data were analyzed with FlowJo software.

For paraffin section of tobacco leaves, the samples were deparaffinized by immersion in Histo-Clear (National Diagnostics, HS2001GLL) and rehydrated through a graded ethanol series. Slides were then fixed with 4% PFA (Thermo Scientific) and washed twice with PBS. Subsequently, slides were treated 1U/μl DNase I (Thermo scientific) at 37 °C for 5 min. The TUNEL assay was then performed using the DeadEnd Fluorometric TUNEL System (Promega, G3250) following the manufacturer’s instructions. Slides were mounted with ProLong Diamond Antifade Mountant with DAPI (Invitrogen, P36966) and cover glasses (Fisherbrand). Imaging was performed using a Leica SP8 confocal microscope, and the intensity of the TUNEL signal was quantified using Fiji software.

### eCLIP-seq

eCLIP-seq in mESCs was performed as previously described^51^ with modifications. mESCs were UV crosslinked at 400 mJ/cm^2^ (254 nm) once and nuclei were isolated following the cell fractionation protocol. Nuclei were lysed in eCLIP lysis buffer (50 mM Tris-HCl pH 7.5, 100 mM NaCl, 1% NP-40, 0.1% SDS, 0.5% sodium deoxycholate, 1× protease inhibitor, 0.5 U/μl Murine Rnase inhibitor) and sonicated using Bioruptor to solubilize the chromatin. The supernatant was partially digested with Rnase I at 37°C for 5 min and quenched by adding SUPERase•In RNase inhibitor. A 10% aliquot of the mixture was set aside for sized-matched input. The remaining mixture was incubated with ALKBH5 or HA antibody pre-bound to Dynabeads Protein A/G (Thermo Scientific) at room temperature for 2 hours. The beads were sequentially washed with high-salt wash buffer (50 mM Tris-HCl pH 7.5, 1M NaCl, 1 mM EDTA, 1% NP-40, 0.1% SDS, 0.5% sodium deoxycholate) and low salt wash buffer (20 mM Tris-HCl pH 7.5, 5 mM NaCl, 10 mM MgCl_2_, 0.2% Tween-20). RNA-protein complexes were dephosphorylated with FastAP (Thermo Scientific, EF0651) and T4 PNK (NEB, M0201L). After ligation with the 3’ RNA adaptor, excessive adaptor was removed with additional washes using high salt wash buffer and low salt wash buffer. Subsequently, IP and Input samples were separated using NuPAGE 4–12% Bis-Tris gels and wet-transferred to a nitrocellulose membrane at 30 V overnight. A region of the membrane corresponding to the expected protein size plus ~75 kDa was excised. RNA fragments were released using Proteinase K (NEB, P8107S) treatment at 37°C for 30 min and purified using the RNA Clean & Concentrator-5 Kit (Zymo, R1013). RNA was reverse transcribed with SR-RT primer using SuperScript III (Thermo Scientific, 18080093), ligated with a 3′ cDNA adaptor, and PCR amplified using LongAmp Taq 2X Master Mix (NEB, M0287L). Fragments between 160 and 307 bp were isolated from a 3.5% low-melt agarose gel. Sequencing was performed on an Illumina NovaSeq X platform in paired-end mode (150 bp per read) at Azenta company. Details of adaptors and primers sequences are provided in Supplementary Table 1.

### *Arabidopsis* caRNA and nuclei isolation

*Arabidopsis* nuclei fractionation was performed as described previously^52^ with modifications. Briefly, 3–5 g of *Arabidopsis* seedlings were frozen in liquid nitrogen and ground into powder using a mortar and pestle. The resulting powder was transferred to 20 ml of Honda buffer (0.44 M sucrose, 1.25% Ficoll, 2.5% Dextran T40, 20 mM Tris-HCl pH 7.5, 10 mM MgCl_2_, 1 mM DTT, 1×protease inhibitor) and incubated on ice for 10 minutes. The mixture was filtered through a 40 μm cell strainer. Following centrifugation at 600 × g for 5 minutes, the pellet was resuspended in 15 ml of Honda buffer containing 0.5% Triton X-100 and incubated on ice for another 5 minutes. The suspension was filtered through two layers of Miracloth (Sigma, 475855) and centrifuged at 600 × g for 10 minutes. The resulting pellet was washed twice with Honda buffer containing 0.1% Triton X-100 and designated as the nuclei fraction for subsequent applications such as ATAC-seq, CUT&Tag, and CUT&RUN. The pellet was resuspended in 300–500 μl of Honda buffer, and a 10 μl aliquot was stained with DAPI to assess the nuclei count under a fluorescence microscope.

For caRNA extraction, the nuclei pellet was washed with cold PBS containing 1 mM EDTA. The nuclei were then resuspended in 200 μl of ice-cold glycerol buffer (20 mM Tris-HCl, pH 7.9, 75 mM NaCl, 0.5 mM EDTA, 0.85 mM DTT, 50% glycerol, 1× protease inhibitor, 0.2 U/μl SUPERase•In RNase inhibitor) with gentle mixing. Two volumes of cold nuclei lysis buffer (10 mM HEPES, pH 7.6, 1 mM DTT, 7.5 mM MgCl₂, 0.2 mM EDTA, 0.3 M NaCl, 1 M urea, 1% NP-40, 1× protease inhibitor, 0.2 U/μl SUPERase•In RNase inhibitor) were added, and the mixture was vortexed vigorously for 5 seconds twice. The lysate was incubated on ice for 2 minutes, and the pellet was collected by centrifugation at 15,000 × g for 2 minutes at 4 °C. The pellet was gently rinsed twice with cold PBS containing 1 mM EDTA and collected as the chromosome-associated fraction. For caRNA extraction, the pellet was resuspended in 200 μl of Turbo DNase I mixture (5 μl Turbo DNase I, 1× Turbo DNase I buffer, 0.2 U/μl SUPERase•In RNase inhibitor) and incubated at 37 °C for 15 minutes. Subsequently, three volumes of TRIzol LS reagent were added for RNA extraction.

### Spike-in calibrated ATAC-seq

Spike-in calibrated ATAC-seq was performed using the ATAC-Seq Kit (Active Motif, 53150) according to the manufacturer’s instructions. For mESCs, 5 × 10^4^ cells were collected, mixed with 10 μl of ATAC-Seq Spike-In Nuclei (Active Motif, 53154), and washed once with ice-cold PBS. The cell pellet was resuspended in 100 μl of ice-cold ATAC Lysis Buffer and centrifuged at 500 × g for 10 minutes. For *Arabidopsis* nuclei, 5 × 10^4^ nuclei were collected, mixed with 10 μl of ATAC-Seq Spike-In Nuclei (Active Motif, 53154), and washed once with ice-cold PBS. The tagmentation reaction was performed following the ATAC-Seq Kit protocol. Immediately after the tagmentation reaction, DNA fragments were purified and subjected to PCR amplification using Q5 High-Fidelity 2X Master Mix (NEB, M0492L). The PCR products were purified with AMPure XP Beads (Beckman Coulter, A63882). Sequencing was carried out on an Illumina NovaSeq X platform in paired-end mode (150 bp per read) at Azenta or the University of Chicago Genomics Facility.

### CUT&Tag

CUT&Tag was performed using the CUT&Tag-IT Core Assay Kit (Active Motif, 53176) according to the manufacturer’s instructions. For mESCs, 2 × 10^5^ cells were collected, mixed with 10 μl of CUT&Tag-IT Spike-In Nuclei (Active Motif, 53168), and washed twice with 1× Wash Buffer. For *Arabidopsis*, 2.5 × 10^5^ isolated nuclei were mixed with 10 μl of CUT&Tag-IT Spike-In Nuclei (Active Motif, 53168) and washed twice with 1× Wash Buffer. For each sample, 20 μl of Concanavalin A bead slurry was used. During cells washing, Concanavalin A beads were activated by incubation in 1× Binding Buffer. mESCs or *Arabidopsis* nuclei were then bound to the activated Concanavalin A beads by incubation in 1× Binding Buffer for 10 minutes. Bound mESCs or *Arabidopsis* nuclei were resuspended in 50 μL of ice-cold Antibody Buffer containing specific primary antibodies and spike-in antibody, followed by overnight incubation at 4 °C. After removing the primary antibodies, the samples were incubated with Guinea Pig Anti-Rabbit secondary antibody (1:100 dilution) in Dig-Wash Buffer at room temperature for 60 minutes. Following three washes with Dig-Wash Buffer, the samples were incubated with CUT&Tag-IT Assembled pA-Tn5 Transposases at room temperature for 60 minutes. The tagmentation reaction was initiated by adding 125 μL of Tagmentation Buffer and incubating at 37 °C for 60 minutes. The reaction was stopped, and DNA fragments were released by adding 4.2 μl of 0.5 M EDTA, 1.25 μl of 10% SDS, and 1.1 μl of Proteinase K (10 mg/ml), followed by incubation at 55 °C for 60 minutes. The DNA fragments were purified and subjected to PCR amplification using Q5 High-Fidelity 2X Master Mix (NEB, M0492L). PCR products were purified with AMPure XP Beads (Beckman Coulter, A63882). Sequencing was carried out on an Illumina NovaSeq X platform in paired-end mode (150 bp per read) at Azenta or the University of Chicago Genomics Facility.

### CUT&RUN

CUT&RUN was performed using the ChIC/CUT&RUN Assay Kit (Active Motif, 53180) according to the manufacturer’s instructions. For histone modifications, YY1, or EP300, CUT&RUN Spike-In Control (Active Motif, 53183) was used as the spike-in. For HA or Flag-tagged proteins, the SNAP-CUTANA HA Tag Panel (Epicypher, 19–5002) or SNAP-CUTANA DYKDDDDK Tag Panel (Epicypher, 19–5001), respectively, was used as the spike-in.

For mESCs, 2.5 × 10^5^ cells were collected and mixed with 10 μl of CUT&RUN Spike-In Nuclei (Active Motif, 53183). mESC nuclei were isolated by incubation with Complete Nuclei Isolation Buffer on ice for 10 minutes. After incubation, the CUT&RUN Spike-In Nuclei were added to mESCs or *Arabidopsis* nuclei and washed twice with Complete Dig-Wash Buffer. During cells washing, Concanavalin A beads were activated by incubation in 1× Binding Buffer. mESC or *Arabidopsis* nuclei were then bound to the activated Concanavalin A beads by incubation in Complete Dig-Wash Buffer for 10 minutes.

The bound nuclei were resuspended in 50 μl of ice-cold Antibody Buffer containing specific primary antibodies and spike-in antibody, followed by overnight incubation at 4 °C. For samples using the SNAP-CUTANA HA Tag Panel or SNAP-CUTANA DYKDDDDK Tag Panel, 1 μl of the corresponding panel was added instead of the spike-in antibody during this step.

The following day, the primary antibody mixture was removed, and the beads were washed twice with Cell Permeabilization Buffer. 50 μl of Cell Permeabilization Buffer containing 2.5 μl of ChIC/CUT&RUN pAG-MNase was added to each sample, followed by a 10-minute incubation at room temperature. After two washes with Cell Permeabilization Buffer, targeted chromatin digestion was activated by the addition of 1 μl of 0.1 M CaCl_2_ and incubation at 4 °C for 2 hours. The reaction was stopped by adding 40 μL of Stop Solution.

The released DNA fragments were purified and used for library preparation with the NEBNext Ultra II DNA Library Prep Kit for Illumina (NEB, E7645S) according to the manufacturer’s instructions. Sequencing was performed on an Illumina NovaSeq X platform in paired-end mode (150 bp per read) at Azenta or the University of Chicago Genomics Facility.

### Nascent RNA-seq

mESCs were seeded into eight 6-cm dishes at equal cell densities one day before EU labeling. The following day, cells were incubated in media containing 1 mM EU for 10, 20, 40, or 60 minutes. After incubation, cells were lysed with TRIzol (Invitrogen), and total RNA was extracted. An equal amount of RNA extracted from EU-labeled Drosophila cells was added to each sample as a spike-in for further normalization.

Nascent RNA was captured using the Click-iT Nascent RNA Capture Kit (Invitrogen, C10365) according to the manufacturer’s instructions. The captured RNA was eluted from the beads using elution buffer (95% v/v formamide, 10 mM EDTA pH 8.0, 1.5 mM biotin) by heating at 65 °C for 5 minutes followed by 90 °C for another 5 minutes. The eluted RNA was purified by ethanol precipitation with glycogen (Invitrogen) as a carrier and dissolved in nuclease-free water (Thermo Scientific).

RNA libraries were constructed using the SMARTer Stranded Total RNA-seq Kit v2 (Takara, 634413) according to the manufacturer’s protocol. Sequencing was performed on an Illumina NovaSeq X platform in paired-end mode (150 bp per read) at Azenta.

### caRNA MeRIP-seq

For mESCs, 1 μl of 1:1000 diluted m^6^A and non-m^6^A spike-in controls from the EpiMark N6-Methyladenosine Enrichment Kit (NEB, E1610S) was added as a spike-in to 1 μg of non-ribosomal RNA isolated from the chromatin-associated fraction. For *Arabidopsis*, 1 μl of 1:100 diluted m^6^A and non-m^6^A spike-in controls from the same kit was added as a spike-in to 200 ng of non-ribosomal RNA isolated from the chromatin-associated fraction. RNA was fragmented using RNA Fragmentation Reagents (Invitrogen) by incubating at 70 °C for 6 minutes, followed by purification with the Oligo Clean & Concentrator Kit (Zymo Research, D4061). 5% of the fragmented RNA was saved as input. m^6^A immunoprecipitation was performed using the EpiMark N6-Methyladenosine Enrichment Kit (NEB) according to the manufacturer’s instructions. Library preparation was carried out using the SMARTer Stranded Total RNA-seq Kit v2 (Takara, 634413) following the manufacturer’s protocol. Sequencing was performed in paired-end mode (150 bp per read) on an Illumina NovaSeq X platform at Azenta or the University of Chicago Genomics Facility.

### Measurement of nuclear RNA lifetime by RT-qPCR

WT and *Alkbh5*-ΔcIDR mESCs were seeded into eight 6-cm dishes at equal cell densities one day prior to the assay. The following day, cells were treated with media containing 5 μg/mL actinomycin D for 0, 3, and 6 hours. After incubation, cells were collected, and nuclear fractions were isolated following the cell fractionation protocol. Nuclear RNA was extracted using TRIzol Reagent (Thermo Scientific). To normalize RNA quantities, 1 μl of 1:100 diluted m^6^A and non-m^6^A spike-in controls from the EpiMark N6-Methyladenosine Enrichment Kit (NEB) was added proportionally to the total nuclear RNA. RNA levels were quantified by RT-qPCR. The half-life (t_1/2_) of nuclear RNA was calculated using a one-phase decay model in GraphPad Prism.

### CLIP-qPCR of YTHDC1

CLIP was performed as described in the eCLIP-seq section, with the following modifications: RNA was partially digested with 1 U/μl RNase T1 (Thermo Scientific) at 25 °C for 5 minutes, followed by chilling on ice for 5 minutes. After immunoprecipitation, the beads were washed sequentially with high-salt and low-salt wash buffers, followed by Proteinase K digestion. RNA was then extracted using TRIzol Reagent (Thermo Scientific) and subjected to RT-qPCR analysis.

### Plant materials and growth conditions

All wild-type (WT) and transgenic *Arabidopsis* lines used in this study were in the *Arabidopsis thaliana* Columbia (Col-0) background. Seeds were surface sterilized in 10% sodium hypochlorite solution for 10 minutes, washed five times with sterile water, and grown on Murashige and Skoog (MS) medium (PhytoTech Labs, M519) supplemented with 1.5% sucrose and 0.8% agar (pH 5.8). Seedlings were grown under controlled conditions in either a growth chamber or a growth room at 21 °C with a 16-hour light/8-hour dark photoperiod.

All wild-type (WT) and transgenic rice lines used in this study were in the Zhonghua11 (ZH11) background. Rice plants were cultivated in greenhouse and experimental fields. For field cultivation, rice plants were grown in standard paddy conditions during normal rice planting season in experimental fields located in Guangzhou, China (N 23°19′53″, E 113°35′06″). Plants were spaced at 20 × 20 cm (row × plant distance) and managed following standard agricultural practices, including irrigation, fertilization, and disease control, in accordance with normal agricultural guidelines.

### Plant transformation

Transgenic *Arabidopsis* lines were generated via Agrobacterium-mediated floral dipping^53^ in the wild-type Col-0 background. Transformed T1 seeds were screened on compound soil watered with BASTA solution. Transgenic lines with successful gene insertion were confirmed by western blotting and basta resistance. T3 homozygous lines were selected and used for all subsequent analyses.

For transgenic rice lines, transformants were selected on medium supplemented with BASTA. Twenty independent T0 lines were generated to obtain T1 progeny. T-DNA copy number (CN) in T1 plants was determined by quantitative PCR (qPCR) using primers specific for the BASTA resistance gene and the internal reference gene *Actin*, following established protocols^54^. At least three independent lines with a single-copy insertion (CN = 1) of the target gene were selected for further analysis and advanced to the T2 generation. Lines with multiple T-DNA insertions were excluded. Homozygous lines in the T2 generation were identified by qPCR-based T-DNA copy number analysis. Primer sequences are provided in Supplementary Table 1.

### Root length assay

Seeds were surface sterilized by immersion in 10% sodium hypochlorite solution for 15 minutes, followed by five washes with deionized water. Sterilized seeds were plated on Murashige and Skoog (MS) medium (PhytoTech Labs) supplemented with 0.8% agar and 1.5% sucrose. To synchronize germination, the plates were kept in the dark at 4 °C for 72 hours. Plates were then positioned vertically at a 90° angle and transferred to a growth chamber with a 16-hour light/8-hour dark photoperiod at 21 °C. After 10 days of growth, root length was quantified by scanning the seedlings and analyzing the images using Fiji software.

### Measurements of photosynthesis parameters

Photosynthetic parameters of WT and transgenic rice lines at the grain-filling stage were measured using a portable photosynthesis measurement CI-340 system (Zealquest Scientific Technology Co., Ltd), following the manufacturer’s instructions. Measurements were conducted on plants grown in both field and greenhouse conditions.

### Agrobacterium-mediated transient expression in tobacco leaves

Agrobacterium-mediated transient expression in *Nicotiana benthamiana* leaves was performed as described^55^. Briefly, *Agrobacterium tumefaciens* strain GV3101 carrying the indicated constructs was cultured overnight at 28°C in LB medium. The bacterial cells were pelleted and resuspended in washing solution containing 10 mM MgCl₂ and 100 μM acetosyringone. The bacterial suspension was diluted to an OD_600_ of 0.5 using infiltration solution (1/4 MS medium supplemented with 1% sucrose, 100 μM acetosyringone, and 0.005% Silwet L-77). Young, fully expanded *Nicotiana benthamiana* leaves were infiltrated with the bacterial suspension using a needleless syringe. Following infiltration, plants were incubated in the dark for 12–24 hours and then transferred to normal growth conditions for a 2-days recovery period. Leaves were then collected for paraffin embedding.

### Paraffin Section Preparation

Paraffin sections were prepared as previously described^56^. Tobacco leaves were fixed in freshly prepared 4% PFA containing 0.1% Tween-20 and 0.1% Triton X-100 at 4°C overnight. To facilitate thorough fixative penetration, tissues were subjected to vacuum infiltration for 15–20 minutes. Following fixation, tissues were dehydrated through a graded ethanol series (30%, 40%, 50%, 60%, 70%, 85%, and 95%), followed by two washes in 100% ethanol to ensure complete dehydration. Samples were then cleared in Histoclear through a stepwise gradient (25%, 50%, 75%, and 100%) and infiltrated with molten paraffin wax at 60°C. Tissues were embedded in paraffin blocks using peel-away molds and allowed to solidify at room temperature. Sections of 7–10 μm thickness were cut using a Leica rotary microtome and floated on sterile water at 42°C for flattening. Slides were dried overnight at 42°C to ensure proper tissue adhesion and stored at room temperature for downstream analyses.

### Chromosome-associated RNA m^6^A-seq and RNA-seq analysis

Raw reads quality control (QC) was performed using fastp^57^. Reads were aligned to either the mouse genome and transcriptome (GRCm39) or the *Arabidopsis* genome and transcriptome (TAIR10), along with spike-in genomes that included unmodified control RNA and m^6^A-modified control RNA (EpiMark N6-Methyladenosine Enrichment Kit, NEB), using HISAT with the parameter “--rna-strandness RF”^58^.

Post alignment, low-quality mapped reads were filtered, and PCR duplicates were removed using Picard. Strand-specific separation of mapped reads was performed using samtools^59^. m^6^A peaks for each strand were identified using MACS2^60^ with the parameters --nomodel and --keep-dup all. Peaks with fewer than 10 reads in the input sample were excluded to minimize bias from expression abundance. Peaks identified in at least two biological replicates were merged using bedtools^61^ for downstream analysis.

For comparison analysis, union peaks across samples were generated using bedtools, and reads under these peaks were counted using featureCounts^62^ for both IP and input samples. Raw counts were normalized to Counts Per Million (CPM). IP enrichment was calculated as CPM_IP_/CPM_Input_. A scaling factor (SF) was computed based on CPM_IP m6A control RNA_/CPM_input m6A control RNA_. The final m^6^A quantification for each sample was calculated using IP enrichment/SF. Pearson correlation coefficients for m^6^A levels across union peaks were calculated to assess reproducibility among biological replicates. Differential m^6^A regions were identified using edgeR^63^, and m^6^A peaks were assigned to genes using ChIPseeker^64^.

Gene and repeats expression levels were quantified by dividing read counts under genes by read counts of the m^6^A control RNA in input samples. Differentially expressed genes (DEGs) were identified using edgeR^63^.

Analyses for different RNA categories, GO enrichment, GSEA, and repeat subfamilies, were performed using clusterProfiler^65^.

### eCLIP-seq data analysis

Raw reads were trimmed using cutadapt with the parameters “-e 0.1 -n 1 -O 6 -q 20 --nextseq-trim=20 -m 21:27 -a NNNNNATCACGAGATCGGAAGAGC -A NNNNNGATCGTCGGACTG”. UMI information was extracted using umi_tools with the parameter “extract --random-seed 1 --bc-pattern=NNNNN --bc-pattern2=NNNNNNNNNNN”.

After QC, clean reads were aligned to the mouse genome (GRCm39) using STAR^66^. Duplicate reads were filtered based on UMI information using umi_tools. Reads mapped to rRNA were removed. Peaks were called using MACS2^60^, and peak distances to genes were calculated using ChIPseeker^64^. For profile visualization and IGV visualization, bigWig files were generated using deepTools^67^ with RPKM normalization.

### Transcription rate data analysis

Data QC and alignment were performed using fastp^57^ and HISAT2^58^, respectively. The counts under genes and repeats were calculated using featureCounts^62^. For each gene, the expression level was normalized by dividing the raw counts under genes by the raw counts under ERCC (Thermo Scientific).

For each time point, the relative transcription rates were normalized to the 10-minute sample. Log_2_ fold changes (log_2_FC) relative to the 10-minute sample were calculated for each time point using edgeR^63^. These log_2_FC values were used to represent the transcription rates for subsequent data analysis.

### CUT&Tag, CUT&RUN, and ATAC-seq analysis

For CUT&Tag, CUT&RUN, and ATAC-seq reads, QC was performed using fastp^57^. Reads were aligned to the mouse or *Arabidopsis* genome and the spike-in sequence using BWA^68^. Low-quality mapped reads were filtered with samtools^59^, and PCR duplicates were removed using Picard. Peaks were called using MACS2. Reads under peaks were calculated using featureCounts^62^.

Normalized signals were calculated as reads under peaks / reads in spike-in sequence. For sample comparisons, edgeR^63^ was used to identify differential peaks based on normalized signals. For profile and IGV visualization, bigWig files were generated using deepTools with the parameters “--scaleFactor SF -bs 10 --normalizeUsing None”, where SF represents 1e6 / reads_spike-in_.

### RNA categories definition

The eRNAs were defined by the union peaks of H3K27ac, H3K4me1, and EP300 in mESCs. The paRNAs were defined by H3K4me3 peaks in mESCs. The *Arabidopsis* RNA categories information was downloaded from the TAIR website.

### Antibodies

The antibodies used in this study are summarized below: DYKDDDDK Tag Monoclonal Antibody Alexa Fluor 488 (Invitrogen, MA1–142-A488, 1:100); Goat anti-Rabbit IgG (H+L) Cross-Adsorbed Secondary Antibody, Alexa Fluor 488 (Invitrogen, A-11008, 1:2000); Goat anti-Mouse IgG (H+L) Cross-Adsorbed Secondary Antibody, Alexa Fluor 488 (Invitrogen, A-11001, 1:2000); Goat anti-Rabbit IgG (H+L) Cross-Adsorbed Secondary Antibody, Alexa Fluor 594 (Invitrogen, A-11012, 1:2000); Goat anti-Mouse IgG (H+L) Cross-Adsorbed Secondary Antibody, Alexa Fluor 594 (Invitrogen, A-11005, 1:2000); HA-Tag (C29F4) Rabbit mAb (Cell Signaling Technology, 3724, 1:1000); Anti-Histone H3 (tri methyl K9) antibody (Abcam, ab8898, 1:1000); Tri-Methyl-Histone H3 (Lys27) (C36B11) Rabbit mAb (Cell Signaling Technology, 9733, 1:1000); Anti-ALKBH5 antibody (Abcam, ab195377, 1:1000); Anti-FTO antibody (Abcam, ab92821, 1:1000); Anti-eIF4A3 antibody (Abcam, ab32485, 1:1000); Recombinant Anti-Mago nashi homolog 2 antibody (Abcam, ab186431, 1:1000); Anti-Aly/Ref antibody (Abcam, ab202894, 1:1000); Anti-Y14 antibody (Abcam, ab5828, 1:1000); Monoclonal ANTI-FLAG M2-Peroxidase (HRP) antibody produced in mouse (Sigma, A8592, 1:2000); Anti-mouse IgG, HRP-linked Antibody (Cell Signaling Technology, 7076S, 1:3000); Anti-rabbit IgG, HRP-linked Antibody (Cell Signaling Technology, 7074S, 1:3000); GAPDH Rabbit mAb (Cell Signaling Technology, 8884S, 1:1000); Mouse monoclonal Anti-Rabbit IgG light chain (HRP) (Abcam, ab99697, 1:2000); Anti-ACT | Actin (AgriSera, AS132640, 1:3000); Histone H3K27ac antibody (Active Motif, 39034, 1:50); Histone H3K9me3 antibody (Active Motif, 39062, 1:50); Histone H3K4me3 antibody (Active Motif, 39060, 1:50); Anti-YTHDC1 antibody (Abcam, ab122340, 2 μg for IP); YY1 (D5D9Z) Rabbit mAb (Cell Signaling Technology, 46395, 1:50); p300 (D8Z4E) Rabbit mAb (Cell Signaling Technology, 86377, 1:50).

### Data availability

High-throughput sequencing data can be accessed at the GEO under accession number GSE287841. Quality control assessments for sequencing data are provided in Supplementary Fig. 1–7. Previously published sequencing data that were reanalyzed in this study are summarized below: ChIP-seq data of CREs in mESCs: GSE29218; Pol II ChIA-PET data in mESCs: ENCSR309NAP and ENCSR067GFU; caRNA MeRIP-seq in *Fto*^−/−^ and WT mESCs: GSE133600; LINE1 ChIRP-seq in mESC: GSE146467; RNA-chromatin interaction data in *Arabidopsis*: GSE163845. Source data are provided with this paper.

### Statistics and reproducibility

Statistical analyses were performed with GraphPad Prism 9 or R (v4.5.2), using either two-tailed Mann-Whitney *U*-tests or unpaired two-tailed t-tests, as specified in the figure legends. No statistical method was used to predetermine sample size. No data were excluded from the analyses. Data distribution was assumed to be normal but this was not formally tested. Microscopy imaging and western blotting were repeated independently three times with similar results.

## Extended Data

**Extended Data Fig.1:**
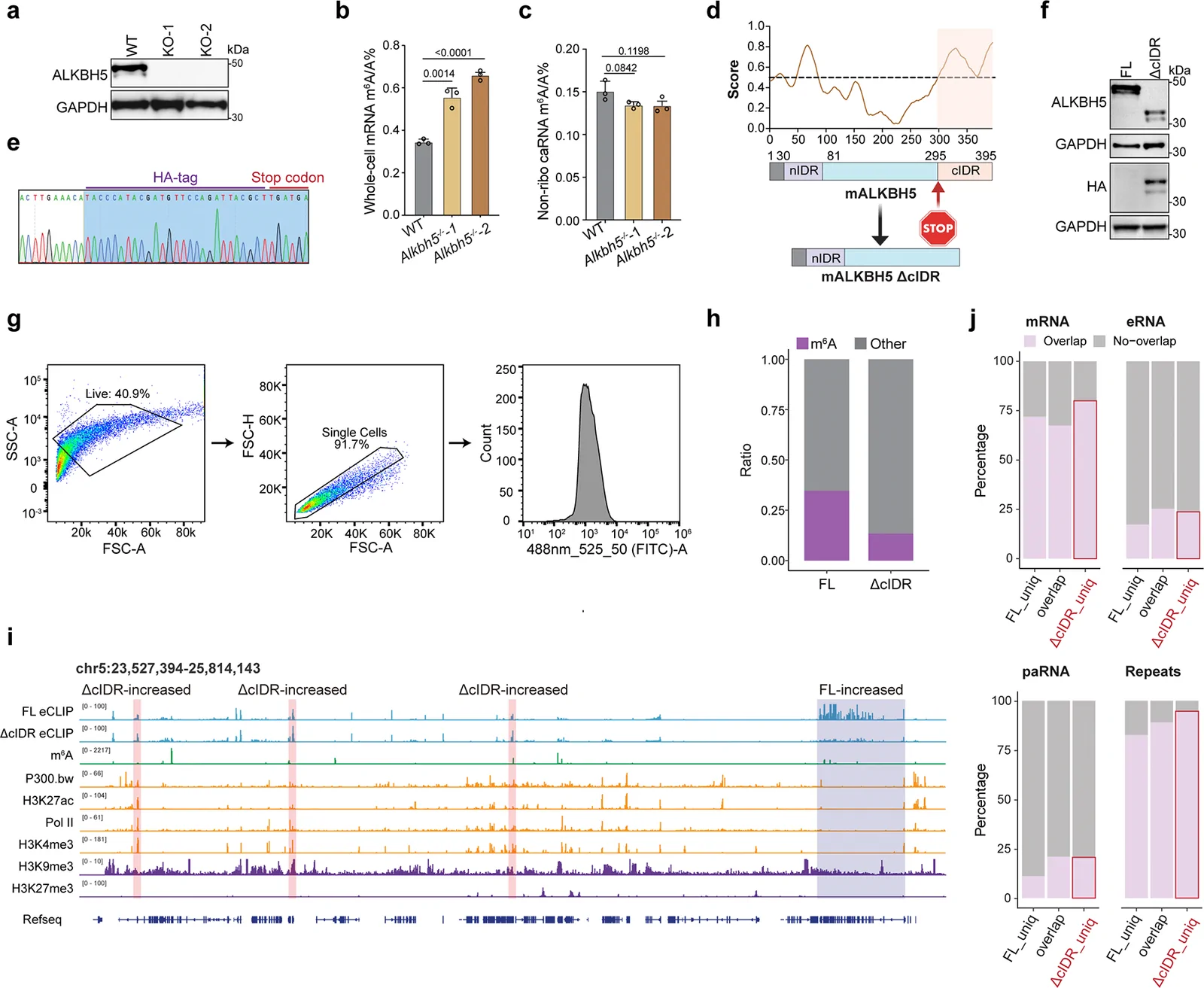
cIDR affects ALKBH5 RNA-binding specificity in mESCs. **a**, Western blot analysis confirming *Alkbh5* knockout in *Alkbh5*^−/−^ mESCs for two independent clones (*Alkbh5* KO-1 and *Alkbh5* KO-2), with GAPDH as a loading control. **b,c**, Quantification of the m^6^A/A ratio by LC-MS/MS in whole-cell polyadenylated RNA (**b**) and non-ribosomal caRNA (**c**) extracted from WT and *Alkbh5*^−/−^ mESCs. Data are shown as mean ± SEM, n = 3 biological replicates. Unpaired two-tailed t tests. **d**, Top: IUPred3 score of mouse ALKBH5 (mALKBH5), where scores greater than 0.5 indicate an IDR. The IDR distribution in human and mouse ALKBH5 is similar. Bottom: schematic diagram of the CRISPR-Cas9 strategy used to delete the cIDR of endogenous ALKBH5 in mESCs by introducing an HA-stop codon upstream of the cIDR-encoding region at the endogenous *Alkbh5* locus. **e**, Representative Sanger sequencing chromatogram confirming successful HA-tagged stop codon knock-in at the targeted *Alkbh5* locus in mESCs. **f**, Western blot analysis of full-length mALKBH5 and mALKBH5-ΔcIDR proteins in WT and *Alkbh5*-ΔcIDR mESCs, respectively. Top: detection of full-length mALKBH5 in WT mESCs and mALKBH5-ΔcIDR in *Alkbh5-*ΔcIDR mESCs. Bottom: confirmation of HA-tag knock-in at the endogenous *Alkbh5* locus in *Alkbh5*-ΔcIDR mESCs. GAPDH was used as a loading control. **g**, Gating strategies for Fig. 1d. **h**, Proportion of eCLIP-seq peak for full-length mALKBH5 (FL) and mALKBH5-ΔcIDR overlapping with m^6^A peaks on mRNA. **i**, Integrated Genomics Viewer (IGV) visualization of chromatin states at differential eCLIP-seq peaks between FL mALKBH5 and mALKBH5-ΔcIDR. Regions enriched in mALKBH5-ΔcIDR are highlighted in red, while regions enriched in FL mALKBH5 are highlighted in blue. **j**, Percentage of eCLIP-seq peaks overlapping with different RNA types. The eCLIP peak categories are the same as in Fig. 1g–i.

**Extended Data Fig.2:**
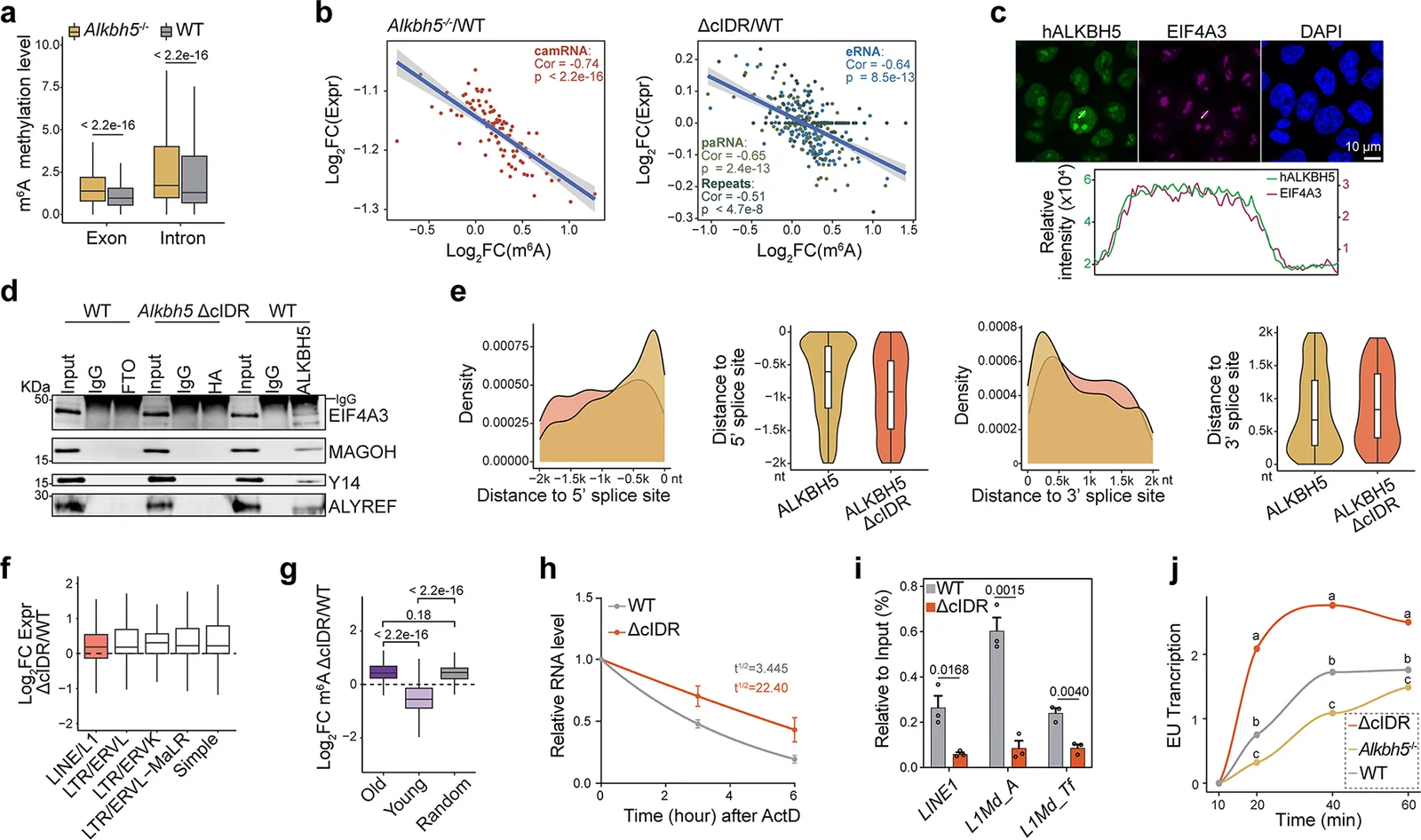
cIDR modulates RNA substrate preference of ALKBH5 in mESCs. **a**, Boxplots showing m^6^A methylation levels of exons and introns in camRNA from WT and *Alkbh5*^−/−^ mESCs. **b**, Scatter plots showing the negative correlation between fold changes in m^6^A levels and expression levels for camRNA upon *Alkbh5*^−/−^ (left) and for carRNA upon *Alkbh5-*ΔcIDR (right). Pearson’s correlation test. **c**, Top: representative immunofluorescence images of hALKBH5 (green) and endogenous EIF4A3 (magenta) in mESCs ectopically expressing Flag-tagged hALKBH5. Nuclei were counterstained with DAPI (blue). Scale bars, 10 μm. Bottom: fluorescence intensity profiles of hALKBH5 (green) and EIF4A3 (magenta) across the indicated lines, showing their colocalization. **d**, Co-immunoprecipitation assays in WT and *Alkbh5*-ΔcIDR mESCs demonstrating that mALKBH5 interacts with components of EJC, while mFTO and HA-tagged mALKBH5-ΔcIDR don’t show such interactions. Interactions of mFTO or mALKBH5 with EJC components were detected in WT mESCs, while interactions of mALKBH5-ΔcIDR with EJC components were detected in *Alkbh5*-ΔcIDR mESCs. **e**, Density of m^6^A sites demethylated by mALKBH5 and mALKBH5-ΔcIDR relative to the nearest splice site. m^6^A sites demethylated by mALKBH5 were defined as hypermethylated sites identified by comparing *Alkbh5*-KO with WT mESCs, and m^6^A sites demethylated by mALKBH5-ΔcIDR were defined as hypomethylated sites identified by comparing *Alkbh5*-ΔcIDR with WT mESCs. Densities were plotted by the distance of m^6^A sites upstream of 5’ splice sites and downstream of 3’ splice sites, respectively. **f**, Fold changes in expression levels of the indicated repeat subfamilies upon *Alkbh5-*ΔcIDR. **g**, Fold changes in m^6^A levels on young and old LINE1 RNAs upon *Alkbh5*-ΔcIDR. "Random" represents repeat RNAs randomly selected across the genome and matched in number to LINE1 RNAs. **h**, Half lifetime of nuclear LINE1 RNA in WT and *Alkbh5*-ΔcIDR mESCs, measured by RT-qPCR after actinomycin D (ActD) treatment. **i**, CLIP-qPCR analysis showing relative enrichment of LINE1 RNA by YTHDC1 in WT and *Alkbh5*-ΔcIDR mESCs. Unpaired two-tailed t test. **j**, Nascent repeat RNA transcription levels over time in WT, *Alkbh5* KO and *Alkbh5*-ΔcIDR mESCs. Data represent mean transcription levels normalized to the 10-minute time point. Different lowercase letters denote statistically significant differences between categories (*p* < 0.05) based on Fisher’s LSD test. For (**a, e-g**), box plots show the median (centre line), IQR (box limits), and whiskers extending to 1.5× IQR. For (**a, g**), *p* values were determined using two-tailed Mann–Whitney *U*-tests. For (**h-i**), data are shown as mean ± SEM. For (**a, e-g, i**), *n* = 3 biological replicates.

**Extended Data Fig.3:**
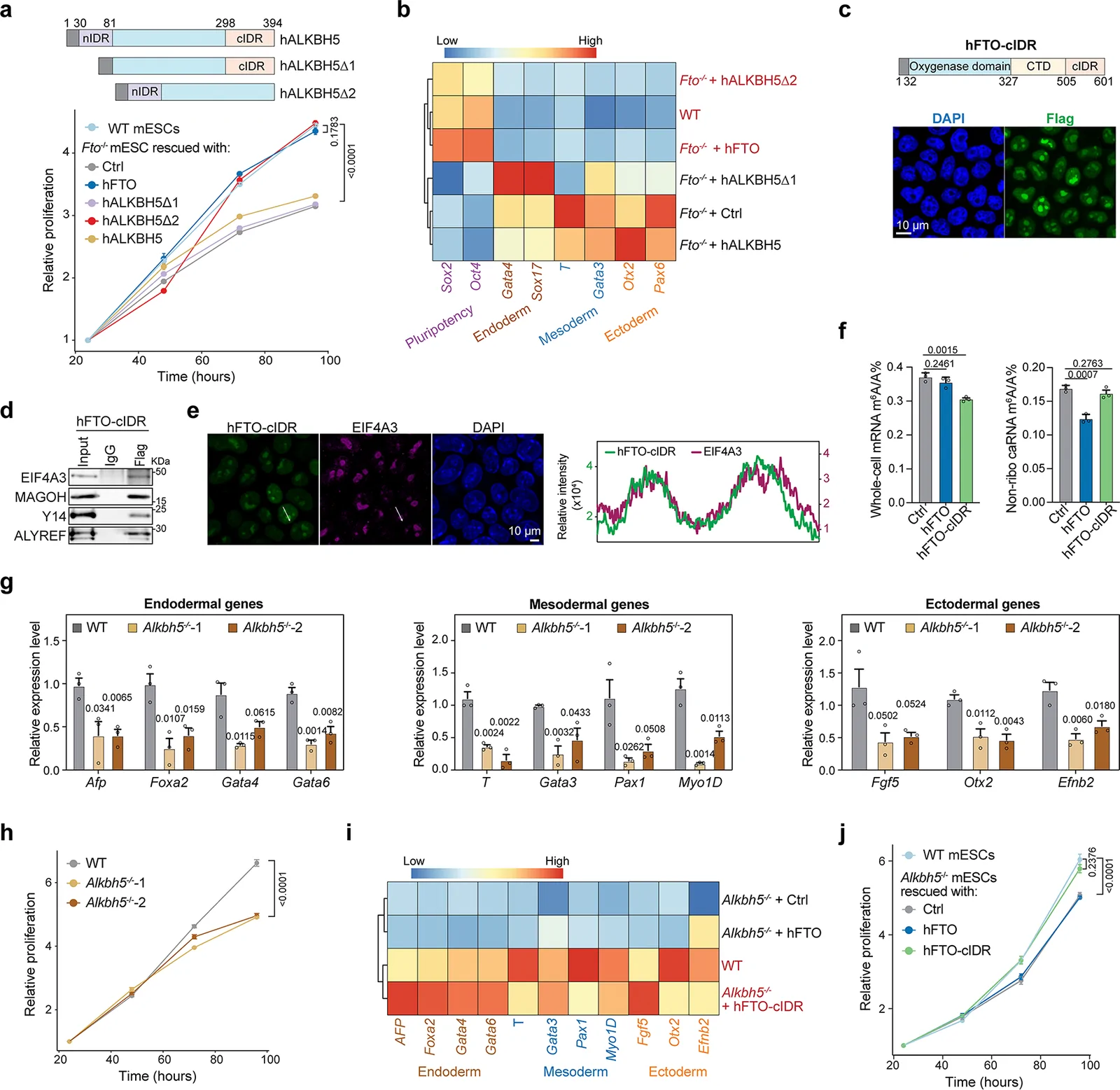
cIDR mediates functional divergence between FTO and ALKBH5 in mESCs. **a**, Top: schematic of human ALKBH5 variants ectopically expressed in *Fto*^−/−^ mESCs. Bottom: growth curves of WT and *Fto*^−/−^ mESCs expressing the indicated variants. *Fto*^−/−^ mESCs transfected with an empty vector served as controls (Ctrl). **b**, Heatmap showing expression levels of differentiation markers in WT mESCs and *Fto*^−/−^ mESCs ectopically expressing an empty vector (Ctrl), human ALKBH5, ALKBH5Δ1 or ALKBH5Δ2 variants. WT mESCs, *Fto*^−/−^ mESCs rescued by human FTO, and *Fto*^−/−^ mESCs rescued by human ALKBH5Δ2 were clustered together. **c**, Top: schematic of the FTO-cIDR fusion variant, where the cIDR of human ALKBH5 is fused to the C-terminus of human FTO. Bottom: representative immunofluorescence images of mESCs expressing Flag-tagged FTO-cIDR. **d**, Co-immunoprecipitation of Flag-tagged hFTO-cIDR ectopically expressed in mESCs, showing interactions with endogenous EJC components. **e**, Left: representative immunofluorescence images of hFTO-cIDR (green) and endogenous EIF4A3 (magenta) in mESCs ectopically expressing Flag-tagged hFTO-cIDR. Nuclei were counterstained with DAPI (blue). Scale bars, 10 μm. Right: fluorescence intensity profiles of hFTO-cIDR (green) and EIF4A3 (magenta) across the indicated lines, showing their colocalization. **f**, LC-MS/MS quantification of the m^6^A/A ratio in whole cell polyadenylated RNA (left) and non-ribosomal caRNA (right) isolated from mESCs ectopically expressing an empty vector (Ctrl), human FTO or FTO-cIDR. **g**, Relative expression levels of differentiation markers in embryoid bodies (EBs) derived from WT and *Alkbh5*^−/−^ mESCs. WT mESCs served as the control, and *p* values represent comparisons to the WT group. **h**, Growth curves showing the reduced proliferation rate of *Alkbh5*^−/−^ mESCs compared to WT mESCs. **i**, Heatmap showing expression levels of differentiation markers in WT mESCs and *Alkbh5*^−/−^ mESCs ectopically expressing an empty vector (Ctrl), human FTO or FTO-cIDR. WT mESCs and *Alkbh5*^−/−^ mESCs rescued by FTO-cIDR were clustered together. **j**, Growth curves of WT and *Alkbh5*^−/−^ mESCs ectopically expressing the indicated variants. *Alkbh5*^−/−^ mESCs transfected with an empty vector served as Ctrl. For (**a, f-h, j**), data are shown as mean ± SEM from three independent experiments and *p* values were determined using unpaired two-tailed t test. For (**a, h, j**), cell numbers at each time point were normalized to cell numbers on day1. Statistical significance was assessed based on cell numbers on the final day.

**Extended Data Fig.4:**
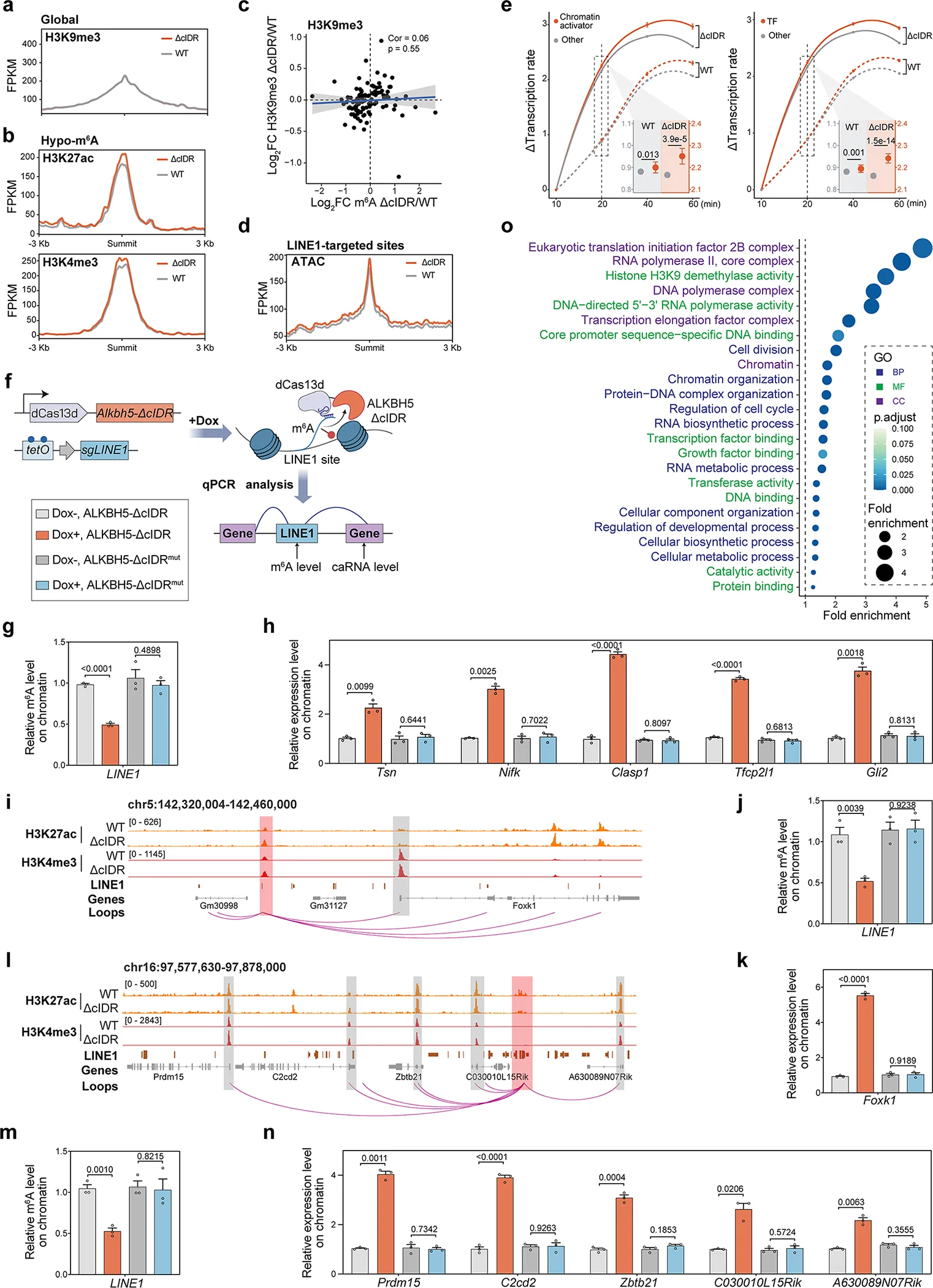
ALKBH5-ΔcIDR mediated m^6^A-hypomethylation promotes chromatin activation in mESCs. **a**, Genome-wide profiles of H3K9me3 in WT and *Alkbh5*-ΔcIDR mESCs,. **b**, Profiles of H3K27ac (top) and H3K4me3 (bottom) in WT and *Alkbh5*-ΔcIDR mESCs at m^6^A-hypomethylated regions, which were differentially identified in *Alkbh5*-ΔcIDR mESCs compared to WT mESCs. **c**, Scatter plots showing the lack of significant correlation between fold changes in m^6^A levels and H3K9me3 levels upon *Alkbh5*-ΔcIDR. **d,** Aggregation plot showing chromatin accessibility in WT and *Alkbh5*-ΔcIDR mESCs within LINE1 RNA-targeted genomic sites. **e**, Transcription rate changes for transcripts annotated as chromatin activators (left) or transcription factors (right, TF) compared with other gene categories in *Alkbh5*-ΔcIDR mESCs and WT mESCs. Dashed lines denote WT mESCs and solid lines denote *Alkbh5*-ΔcIDR mESCs. Insets show the magnified view of transcription rate differences in *Alkbh5*-ΔcIDR and WT mESCs at the 20-min time point following EU labeling of nascent RNA. Two-tailed Mann–Whitney *U*-tests; points represent the medians; error bars represent SEM. **f**, Schematic of a CRISPR-dCas13d system for acute tethering of ALKBH5-ΔcIDR (wild-type or catalytically inactive mutant) to LINE1 RNA. mESCs stably express dCas13d fused to either wild-type ALKBH5-ΔcIDR or its inactive mutant, together with a guide RNA targeting LINE1 RNA under a doxycycline (dox)-responsive Tet operator. Site-specific demethylation of LINE1 RNA was assessed by MeRIP-qPCR, and expression levels of LINE1-looped genes were analyzed by RT-qPCR in the chromatin-associated fraction. (**g, j, m**) Relative m^6^A enrichment on targeted LINE1 RNA in the chromatin-associated fraction measured by MeRIP-qPCR. **g**, **j** and **m** correspond to the loci shown in Fig. 3i, Extended Data Fig. 4i and l, respectively. (**i, l**) Genome browser views showing representative examples of ALKBH5-ΔcIDR-mediated hypomethylated LINE1 RNA-related loops. m^6^A-hypomethylated LINE1 regions are highlighted in red, and promoter regions are highlighted in gray. (**h**, **k**, **n**) Relative expression levels of LINE1 RNA-looped genes in the chromatin-associated fraction measured by RT-qPCR. **h**, **k** and **n** correspond to the loci shown in Fig. 3i, Extended Data Fig. 4i and l, respectively. For (**g, h, j, k, m, n**), data are shown as means ± SEM and p values were calculated using unpaired two-tailed t tests. **o**, GO analysis of upregulated genes upon *Alkbh5*-ΔcIDR. For (**e, g-h, j-k, m-n**), *n* = 3 biological replicates.

**Extended Data Fig. 5:**
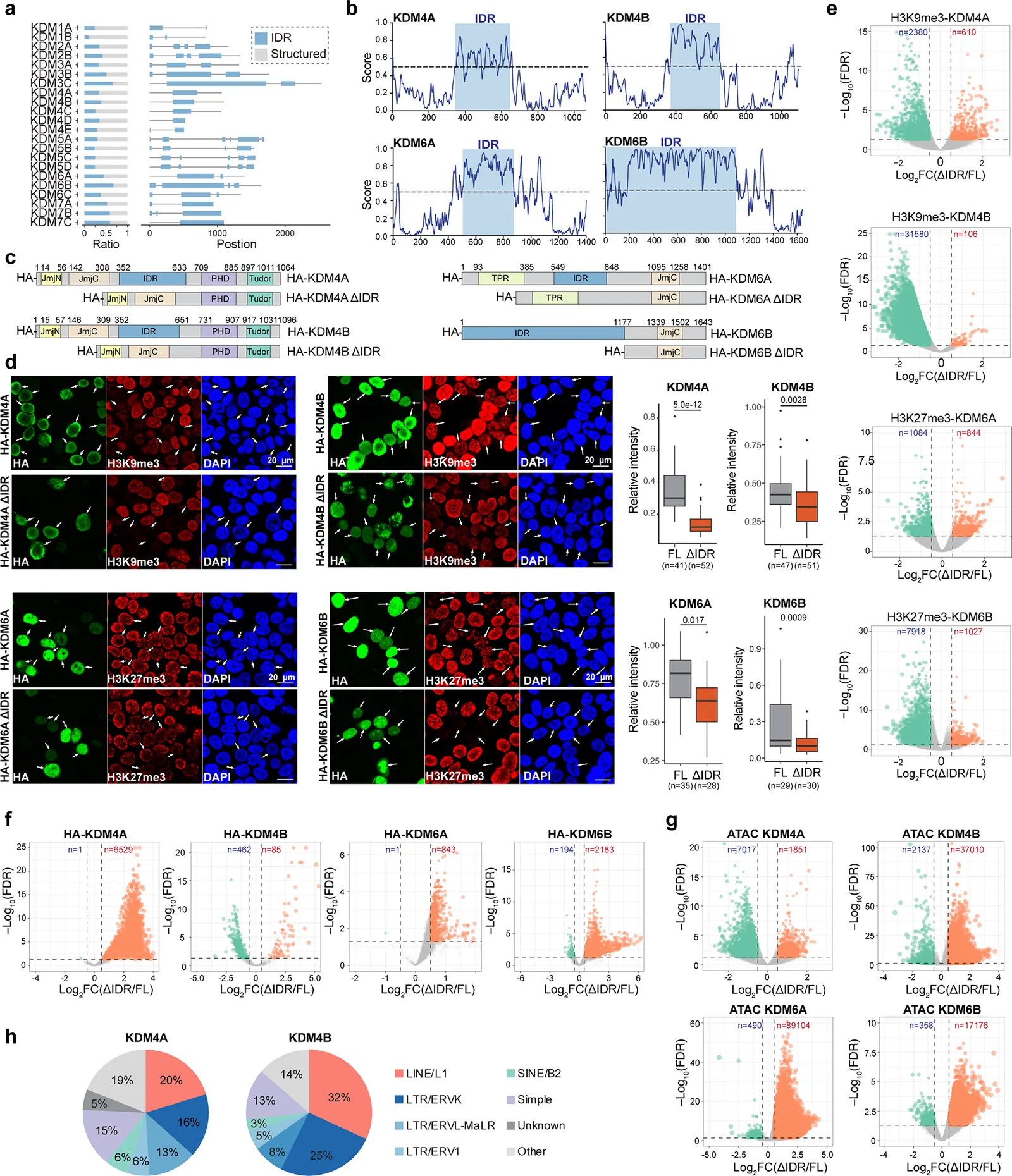
Deletion of IDRs in gene-activating histone demethylases expands the removal of repressive histone modifications. **a**, Prediction of IDRs ratios (left) and positions (right) within human KDM proteins. In the right panel, the x-axis represents the positions of amino acids. **b**, Prediction of IDRs in human KDM4A, KDM4B, KDM6A and KDM6B protein sequences using IUPred3. A score greater than 0.5 indicates the presence of an IDR. **c**, Schematic of HA-tagged human KDMs variants ectopically expressed in HEK293T cells and mESCs. **d**, H3K9me3 demethylation activity of KDM4A/4B variants and H3K27me3 demethylation activity of KDM6A/6B variants in HEK293T cells. Left: representative immunofluorescence images were selected from three independent experiments. Arrowheads indicate transfected cells; non-transfected cells served as controls. Scale bars, 20 μm. Right: quantification of relative H3K9me3 (KDM4A/4B) and H3K27me3 (KDM6A/6B) intensities in transfected cells. Box plots show the median (centre line), IQR (box limits), and whiskers extending to 1.5× IQR; outliers plotted individually. Unpaired two-tailed t tests. **e**, Volcano plots showing changes in H3K9me3 and H3K27me3 levels in mESCs expressing ΔIDR versus full-length variants of KDM4A/4B and KDM6A/6B, respectively. **f**, Volcano plots showing differential HA-tag CUT&RUN signals in mESCs expressing ΔIDR versus full-length variants for KDM4A/4B and KDM6A/6B, respectively. **g**, Volcano plots showing differential ATAC-seq peaks in mESCs expressing ΔIDR versus full-length variants of KDM4A/4B and KDM6A/6B, respectively. **h**, Proportion of downregulated H3K9me3 peaks across TE subfamilies in mESCs expressing ΔIDR versus full-length variants of KDM4A (left) or KDM4B (right).

**Extended Data Fig. 6:**
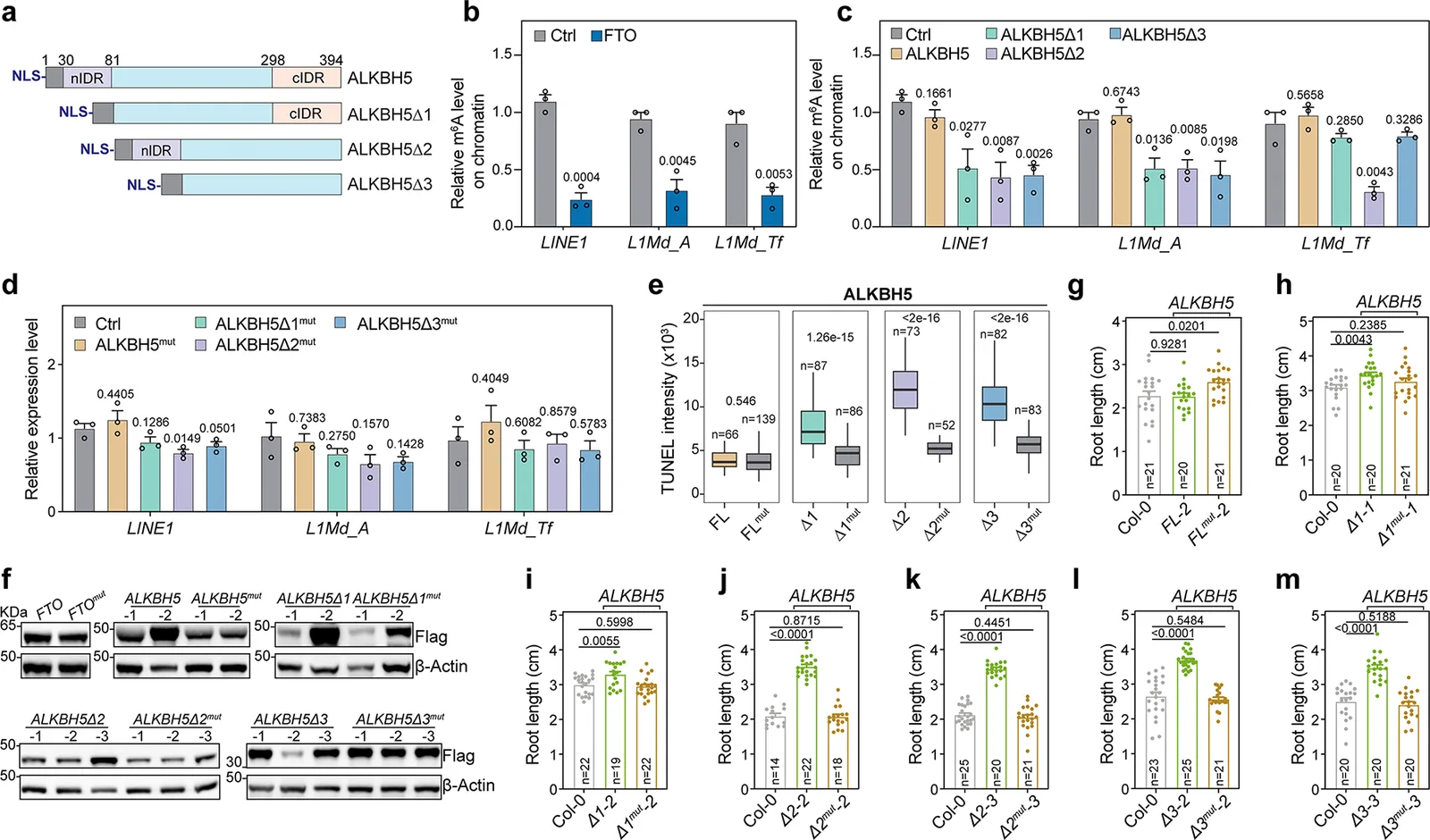
Engineered IDR-deleted human ALKBH5 variants enhance *Arabidopsis* root growth. **a**, Schematics of human ALKBH5 variants ectopically expressed in mESCs, transiently infiltrated into tobacco leaves, and stably transformed into *Arabidopsis*. **b-c,** Relative m^6^A enrichment on chromatin-associated total LINE1 RNA and young LINE1 subfamilies RNAs measured by MeRIP-qPCR in mESCs ectopically expressing human FTO (**b**) or ALKBH5 variants (**c**). **d**, Relative expression levels of whole-cell total LINE1 RNA and young LINE1 subfamilies RNAs measured by RT-qPCR in mESCs ectopically expressing catalytically inactive human ALKBH5 variants (H204A). For **b-d**, empty vector-transfected mESCs served as controls (Ctrl). **e**, DNase I–treated TUNEL assays showing that chromatin opening induced by IDR-deleted human ALKBH5 variants in tobacco leaves is m^6^A-dependent. Box plots show the median (centre line), IQR (box limits), and whiskers extending to 1.5× IQR. TUNEL fluorescence intensity data for FL, Δ1, Δ2 and Δ3 derived from Fig. 5e. **f**, Western blot analysis showing the expression of Flag-tagged human ALKBH5 variants and FTO in *Arabidopsis* transgenic lines. β-Actin was used as a loading control. **g-m**, Primary root length measurements of 10-day-old *Arabidopsis* lines expressing the indicated ALKBH5 variants. Data are shown as means ± SEM (**b-d, g-m**) and *p* values were determined using unpaired two-tailed t tests (**b-e, g-m**). For (**b-d**), *n* = 3 biological replicates. For (**d-e, g-m**), ALKBH5^mut^, ALKBH5Δ1^mut^, ALKBH5Δ2^mut^ and ALKBH5Δ3^mut^ indicate variants with H204A mutation.

**Extended Data Fig. 7:**
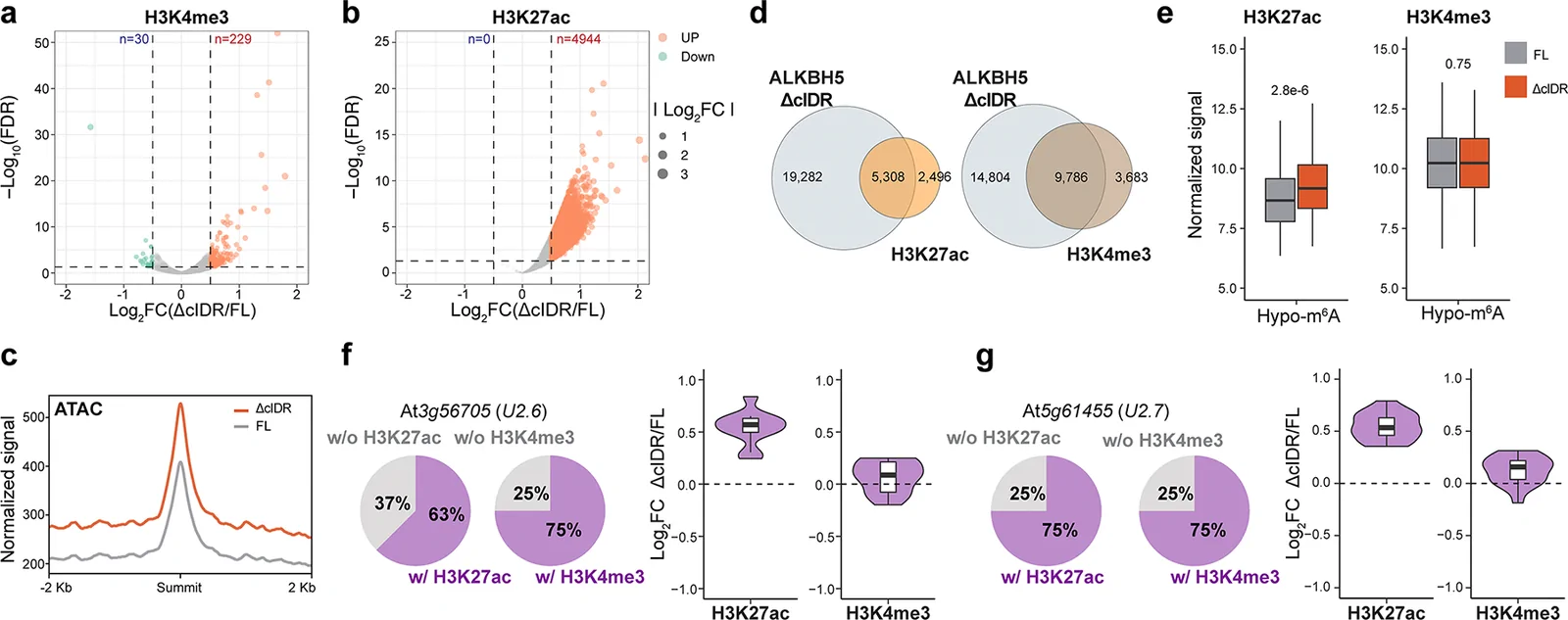
cIDR-deleted ALKBH5 promotes chromatin activation in *Arabidopsis*. **a, b**, Volcano plots showing differential changes in H3K4me3 (a) and H3K27ac (b) signals in *ALKBH5*-ΔcIDR-*Arabidopsis* versus *ALKBH5*-*Arabidopsis*. **c**, Genome-wide profiles of ATAC-seq signals in *ALKBH5*- and *ALKBH5*-ΔcIDR-*Arabidopsis*. **d**, Overlap between Flag CUT&RUN peaks and H3K27ac-marked regions (left) or H3K4me3-marked regions (right) in Flag-tagged *ALKBH5*-ΔcIDR-*Arabidopsis*. **e**, Boxplots showing H3K27ac (left) and H3K4me3 (right) signals at hypomethylated m^6^A regions (Hypo-m^6^A) in *ALKBH5*-ΔcIDR- and *ALKBH5*-*Arabidopsis*, respectively. Hypomethylated m^6^A regions were identified by comparing *ALKBH5*-ΔcIDR- to *ALKBH5*-*Arabidopsis*. Two-tailed Mann–Whitney *U*-tests. **f**, **g**, Chromatin state analysis at At*3g56705* (*U2.6*) and At*5g61455* (*U2.7*) interacted sites in *ALKBH5*- and *ALKBH5*-ΔcIDR-*Arabidopsis*. Top: proportion of At*3g56705* (*U2.6*) and At*5g61455* (*U2.7*) interacted sites marked with H3K27ac or H3K4me3. Bottom: fold changes in H3K27ac or H3K4me3 signals at At*3g56705* (*U2.6*) and At*5g61455* (*U2.7*) interacted sites in *ALKBH5-*ΔcIDR- versus *ALKBH5*-*Arabidopsis*. For (**e-g**), box plots show the median (centre line), IQR (box limits), and whiskers extending to 1.5× IQR, *n* = 3 biological replicates. For **a-g**, FL represents full-length ALKBH5, ΔcIDR represents ALKBH5-ΔcIDR.

**Extended Data Fig. 8:**
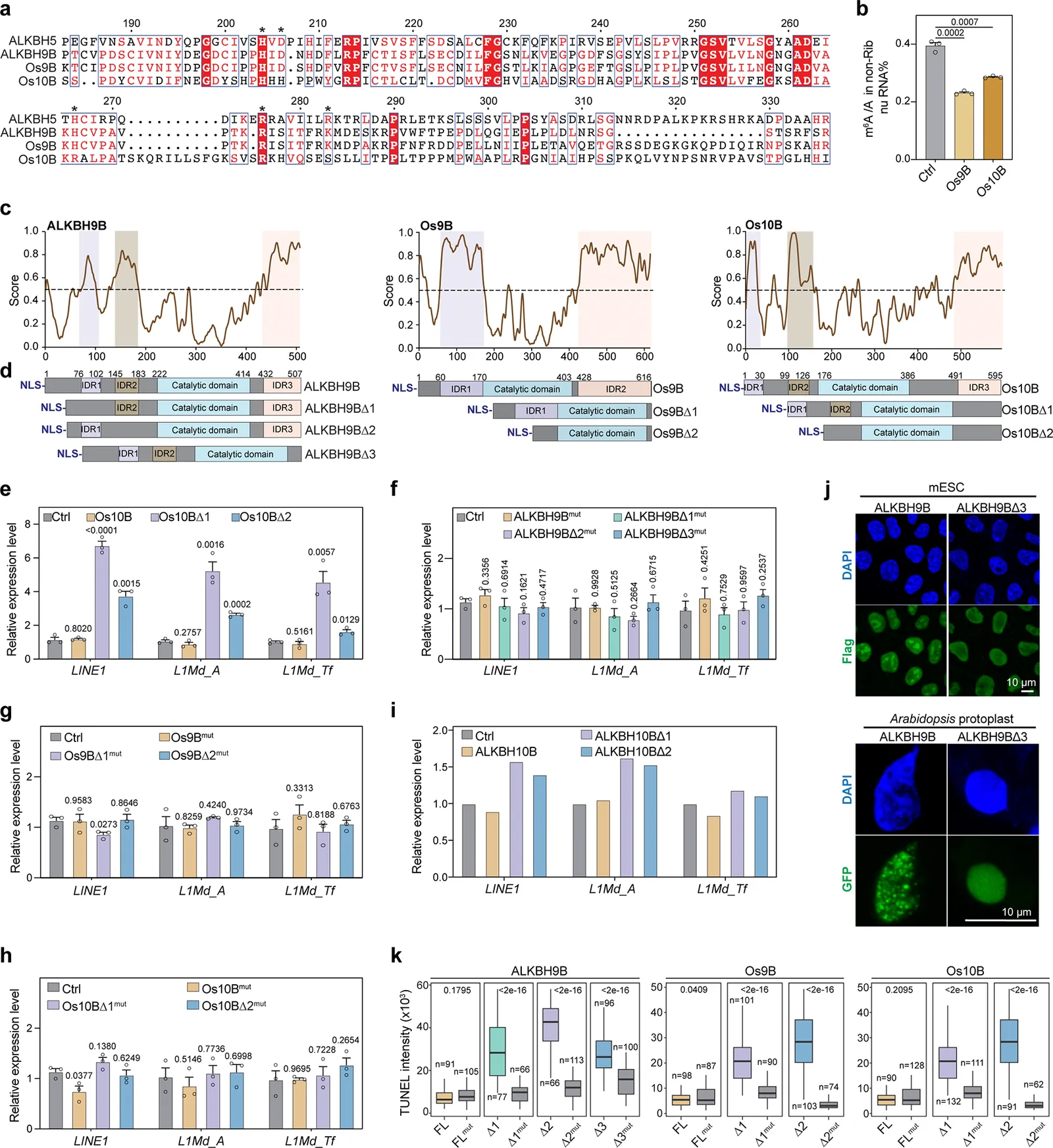
Engineering *Arabidopsis* and rice ALKBH5 orthologs to induce chromatin opening. **a**, Amino acid sequence alignment of human ALKBH5, *Arabidopsis* ALKBH9B, and rice orthologs Os9B and Os10B. Identical residues are boxed in red, and similar residues are shown in red text. Blue frames highlight regions of similarity across different groups. Conserved catalytic residues are marked with asterisks (*). **b**, LC-MS/MS quantification of the m^6^A/A ratio in non-ribosomal RNA extracted from the soluble nuclear fraction (nuRNA) in mESCs ectopically expressing empty vector (Ctrl), rice Os9B or rice Os10B. **c**, Prediction of IDRs in ALKBH9B, Os9B and Os10B using IUPred3. A score greater than 0.5 indicates the presence of an IDR. **d**, Schematic of ALKBH9B, Os9B, and Os10B variants that were ectopically expressed in mESCs, transiently infiltrated into tobacco leaves, and stably transformed into *Arabidopsis*. **e-i**, Relative expression levels of whole-cell total LINE1 RNA and young LINE1 subfamilies RNAs measured by RT-qPCR in mESCs ectopically expressing rice Os10B variants (**e**), catalytically inactive (H335A/D337A mutation) *Arabidopsis* ALKBH9B variants (**f**), catalytically inactive (H324A/D326A mutation) rice Os9B variants (**g**), catalytically inactive (H308A/H310A mutation) rice Os10B variants (**h**), and *Arabidopsis* ALKBH10B variants (**i**). For **i**, ALKBH10BΔ1 refers to deletion of residues 131–190, ALKBH10BΔ2 refers to deletion of residues 501–569. **j**, Representative immunofluorescence images of mESCs and *Arabidopsis* protoplast expressing NLS-fused Flag-tagged (top) or GFP-tagged (bottom) full-length ALKBH9B and cIDR-deleted ALKBH9B (ALKBH9BΔ3), respectively. In mESCs, Flag-tagged ALKBH9B variants were detected by anti-Flag immunostaining (green). In *Arabidopsis* protoplast, GFP-tagged ALKBH9B variants were visualized by GFP fluorescence (green). Nuclei were counterstained with DAPI (blue). Scale bars, 10 μm. **k**, DNase I–treated TUNEL assays showing that chromatin opening induced by IDR-deleted ALKBH9B, Os9B or Os10B variants in tobacco leaves is m^6^A-dependent. Box plots show the median (centre line), IQR (box limits), and whiskers extending to 1.5× IQR. TUNEL fluorescence intensity data for ALKBH9B (FL, Δ1-Δ3) and Os9B (FL, Δ1 and Δ2) derived from Fig. 7c–d. FL represents full-length. For (**b, e-h**), data are shown as means ± SEM, *n* = 3 biological replicates. For (**b, e-h, k**), *p* values were determined using unpaired two-tailed t tests.

**Extended Data Fig. 9:**
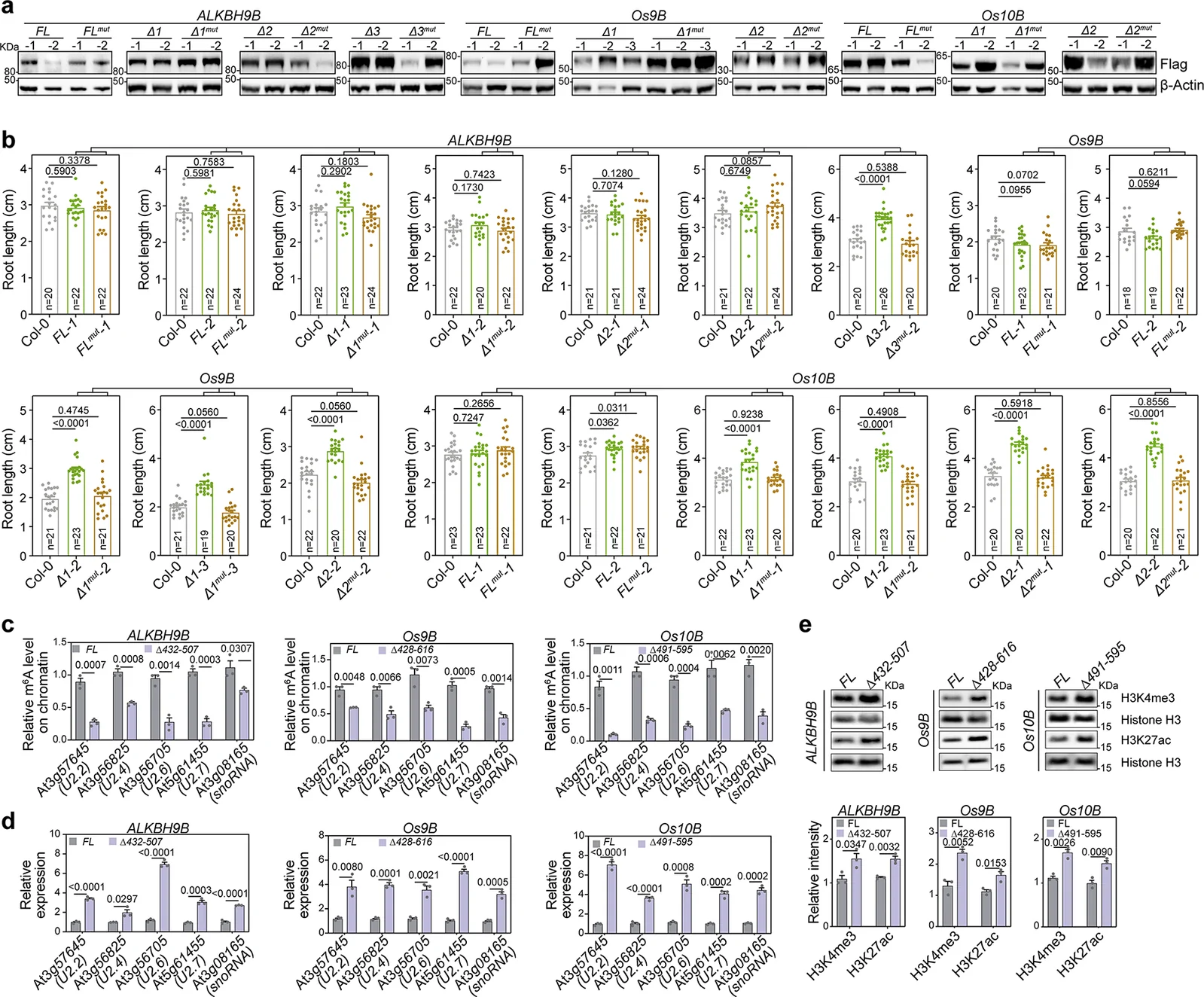
Engineered *Arabidopsis* and rice ALKBH5 orthologs enhance plant growth by demethylating carRNAs and activating chromatin state. **a,** Western blot analysis showing the expression of GFP-Flag-tagged ALKBH9B variants, as well as Flag-tagged Os9B and Os10B variants in *Arabidopsis* transgenic lines. A GFP tag was added to enhance ALKBH9B expression levels. β-Actin was used as a loading control. **b**, Primary root length measurements of 10-day-old *Arabidopsis* lines expressing the indicated ALKBH9B, Os9B, or Os10B variants. **c**, MeRIP-qPCR analysis of relative m^6^A methylation levels of chromatin-associated non-coding RNAs in transgenic *Arabidopsis* lines expressing full-length or cIDR-deleted variants of ALKBH9B, Os9B and Os10B. **d**, Relative expression levels of chromatin-associated non-coding RNAs in transgenic *Arabidopsis* lines expressing full-length or cIDR-deleted variants of ALKBH9B, Os9B and Os10B. **e**, Western blot analysis of H3K4me3 and H3K27ac levels in transgenic *Arabidopsis* lines expressing full-length or cIDR-deleted variants of ALKBH9B, Os9B and Os10B. Histone H3 was used as a loading control. For **c-e**, *n* = 3 biological replicates. For **b-e**, data are shown as mean ± SEM, *p* values were determined using unpaired two-tailed t tests.

**Extended Data Fig. 10:**
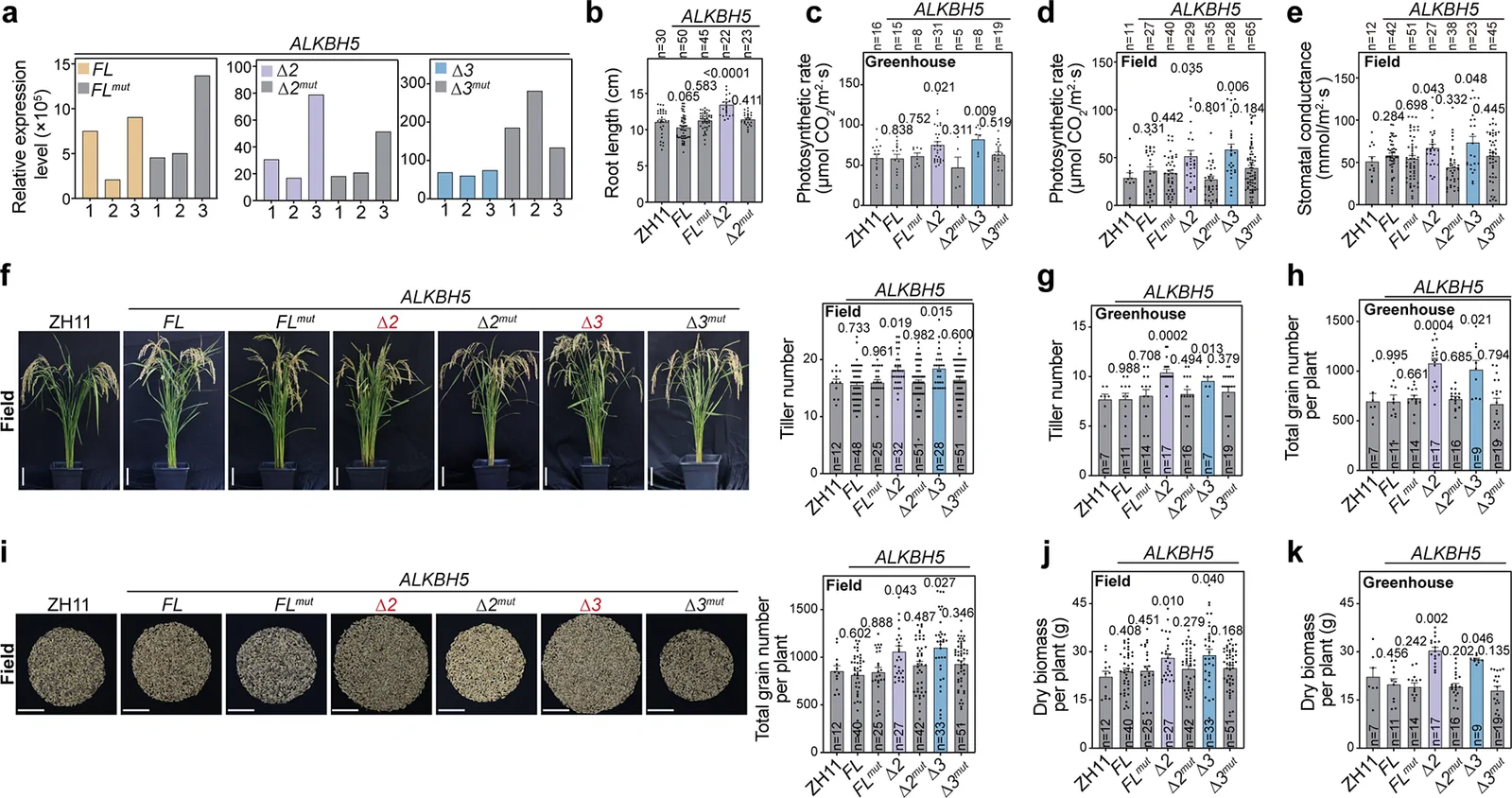
Transgenic expression of engineered cIDR-deleted human ALKBH5 variants in rice increases yield and biomass under both greenhouse and field conditions. **a**, Relative expression levels of human ALKBH5 variants in three independent transgenic rice lines, measured by RT-qPCR. WT ZH11 served as the control. **b**, Root length measurements of 15-day-old WT (ZH11) and transgenic rice seedlings expressing human ALKBH5 variants grown under hydroponic culture conditions. **c-k**, Agronomic traits of WT (ZH11) and transgenic rice lines expressing human ALKBH5 variants grown in the greenhouse (**c, g-h, k**) and field (**d-f, i-j**). **c-d**, Photosynthetic rate measurements. **e**, Stomatal conductance rate measurements. **f-g**, Tiller number per plant. Representative images are shown. Scale bars, 10 cm. **h-i**, Total grain number per plant. Representative images are shown. Scale bars, 4 cm. **j-k**, Dry biomass per plant. For (**b-k**), data are shown as means ± SEM. Unpaired two-tailed t tests against ZH11 group. For (**b, g-h, k**), data for ZH11, *ALKBH5-*, *ALKBH5Δ2-* and *ALKBH5Δ2*^*mut*^*-*rice derived from Fig. 7h–k. For (**a-k**), truncation variants of *ALKBH5* correspond to specific residue deletions, as detailed in Extended Data Fig. 6a. *ALKBH5*^mut^, *ALKBH5*Δ2^mut^ and *ALKBH5*Δ3^mut^ indicate variants with H204A mutation. FL represents full-length.

## Supplementary Material

Supplementary_Table1

## Figures and Tables

**Fig. 1:**
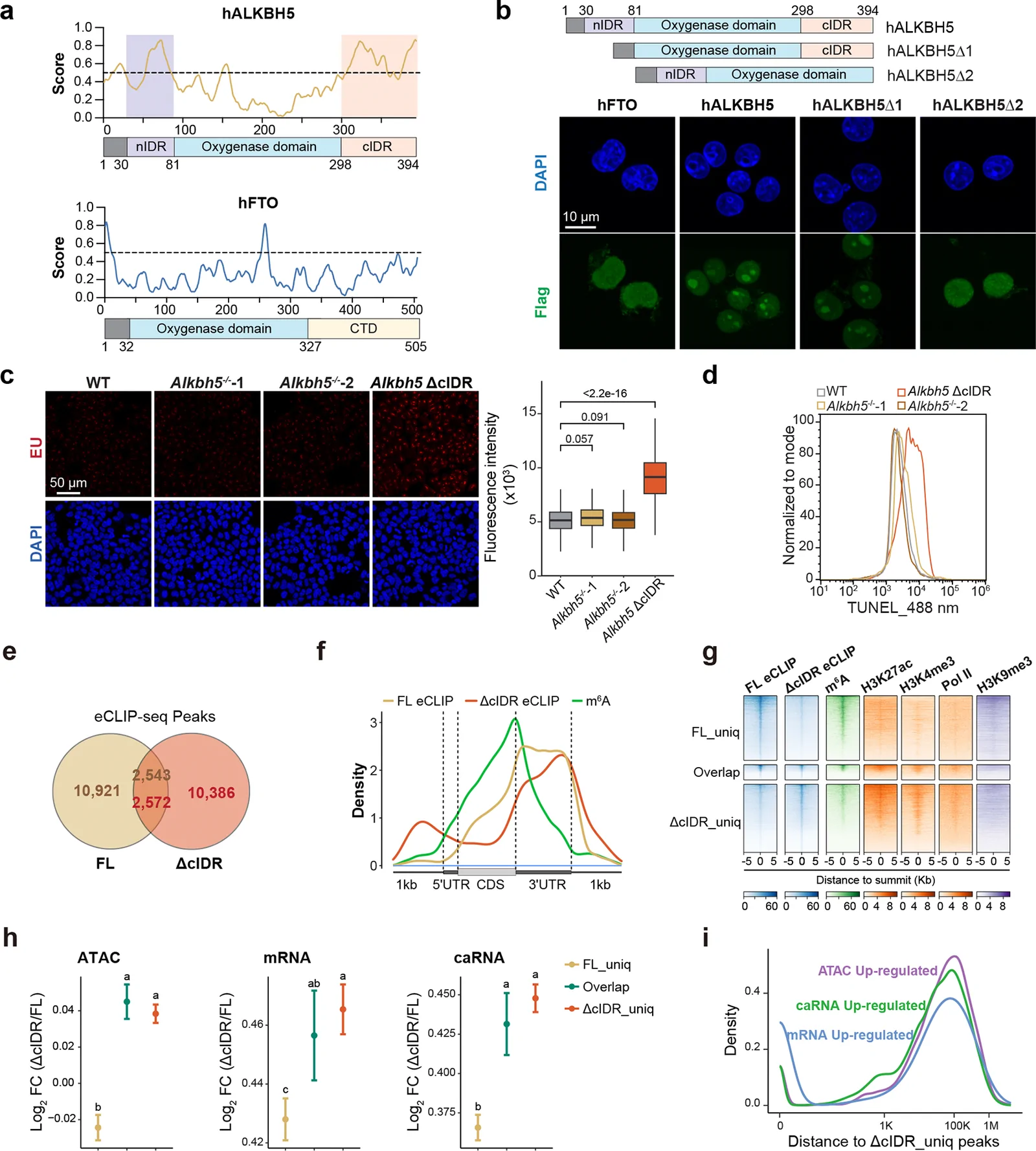
Deletion of ALKBH5 cIDR induces open chromatin and transcription upregulation in mESCs. **a,** Prediction of IDRs in human ALKBH5 and FTO using IUPred3. Score greater than 0.5 indicate IDR. **b,** Schematics of Flag-tagged human ALKBH5 variants and representative immunofluorescence images of mESCs expressing the indicated constructs. Scale bars, 10 μm. **c,** Nascent RNA synthesis in WT, *Alkbh5*^−/−^ and *Alkbh5*-ΔcIDR mESCs measured by 5-ethynyl uridine (EU) incorporation. Scale bars, 50 μm. Six random fields were imaged per condition. Box plots show the median (centre line), interquartile range (IQR, box limits), and whiskers extending to 1.5× IQR. *P* values were determined using unpaired two-tailed t tests. **d,** Chromatin accessibility assessed by TUNEL signals. For **c-d**, *Alkbh5*^−/−^-1 and *Alkbh5*^−/−^-2 refer to two independently generated knockout clonal cell lines. **e**, Overlap of eCLIP-seq peaks between full-length (FL) mALKBH5 in WT mESCs and mALKBH5-ΔcIDR in *Alkbh5*-ΔcIDR mESCs. **f**, Metagene distributions of eCLIP-seq peaks for FL mALKBH5 and mALKBH5-ΔcIDR, along with m^6^A signals in WT mESCs across protein-coding genes. **g,** Heatmaps showing m^6^A levels, Pol II occupancy, and histone modifications across eCLIP peak categories. **h,** Chromatin accessibility and transcriptional changes across eCLIP peak categories, represented as log_2_ fold change (FC) in *Alkbh5*-ΔcIDR mESCs expressing ALKBH5-ΔcIDR relative to WT mESCs expressing full-length ALKBH5 (ΔcIDR/FL). Points represent the median, and error bars represent SEM. Different letters (a, b) denote statistically significant differences between categories (*p* < 0.05) based on Fisher’s Least Significant Difference (LSD) test. For **g-h**, the eCLIP peaks were classified into three groups: peaks unique to full-length mALKBH5 (FL_uniq), overlapping peaks (overlap), and peaks unique to mALKBH5-ΔcIDR (ΔcIDR_uniq). **i,** Frequency distribution of upregulated ATAC, caRNA, and mRNA signals in *Alkbh5*-ΔcIDR mESCs relative to the nearest ΔcIDR-unique peaks. For (**c, h**), n = 3 biological replicates.

**Fig. 2:**
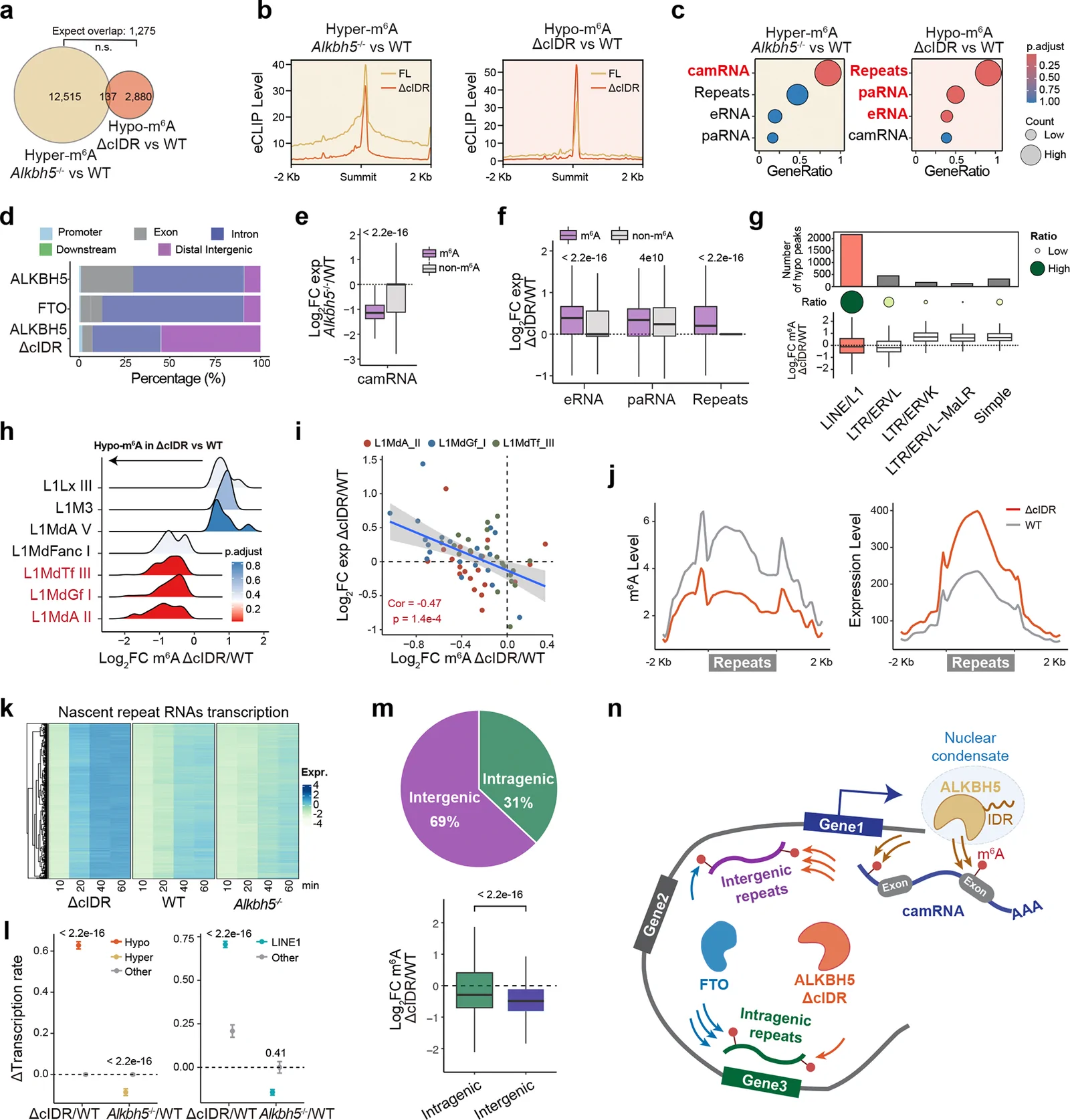
cIDR deletion redirects mALKBH5 from mRNA to chromatin-associated repeat RNAs in mESCs. **a**, Overlap of differentially methylated m^6^A regions (DMRs) between conditions. One-sided Fisher’s exact test. Expected overlap is indicated. n.s., not significant. **b,** Aggregation plots showing eCLIP-seq signals for FL mALKBH5 and mALKBH5-ΔcIDR across indicated regions. **c,** Enrichment analysis of DMRs across RNA categories (two-sided over-representation tests). **d**, Genomic distribution of hypermethylated m^6^A peaks for FTO (*Fto*^−/−^ vs WT mESCs, GSE133600) and ALKBH5 (*Alkbh5*^−/−^ vs WT mESCs), and hypomethylated m^6^A peaks for ALKBH5-ΔcIDR (*Alkbh5*-ΔcIDR vs WT mESCs). **e, f**, Boxplots showing expression fold changes of m^6^A-marked and non-m^6^A-marked camRNA upon *Alkbh5*^−/−^ (**e**), and carRNA upon *Alkbh5*-ΔcIDR (**f**). **g**, Summary of hypomethylated repeat RNAs across subfamilies upon *Alkbh5*-ΔcIDR. **h**, GSEA illustrating the enrichment of DMRs within repeat subfamilies upon *Alkbh5*-ΔcIDR. **i**, Correlation between changes in m^6^A levels and expression levels of enriched LINE1 subfamilies upon *Alkbh5*-ΔcIDR. Pearson’s correlation test. **j,** Aggregation plots showing m^6^A and expression levels of repeat RNAs upon *Alkbh5*-ΔcIDR within mFTO regulatory regions, which were defined as hypermethylated m^6^A peaks upon *Fto*^−/−^ in previous studies^2^. **k,** Heatmap showing nascent repeat RNAs transcription levels over time. **l,** Transcription rate changes of repeat RNAs at hypomethylated regions upon *Alkbh5*-ΔcIDR and hypermethylated regions upon *Alkbh5* KO (left), and comparison between LINE1 and other repeats (right). Points represent the medians; error bars represent SEM. **m**, Proportion of hypomethylated LINE1 RNAs and m^6^A-level changes of LINE1 RNAs in intergenic versus intragenic regions upon *Alkbh5*-ΔcIDR. **n**, Model illustrating distinct substrate specificities of ALKBH5, FTO and ALKBH5-ΔcIDR on chromatin. For (**e-f, l-m**), two-tailed Mann–Whitney *U*-tests. For (**e-g, m**), box plots show the median (centre line), IQR (box limits), and whiskers extending to 1.5× IQR. For (**e-g, l, m**), n = 3 biological replicates.

**Fig. 3:**
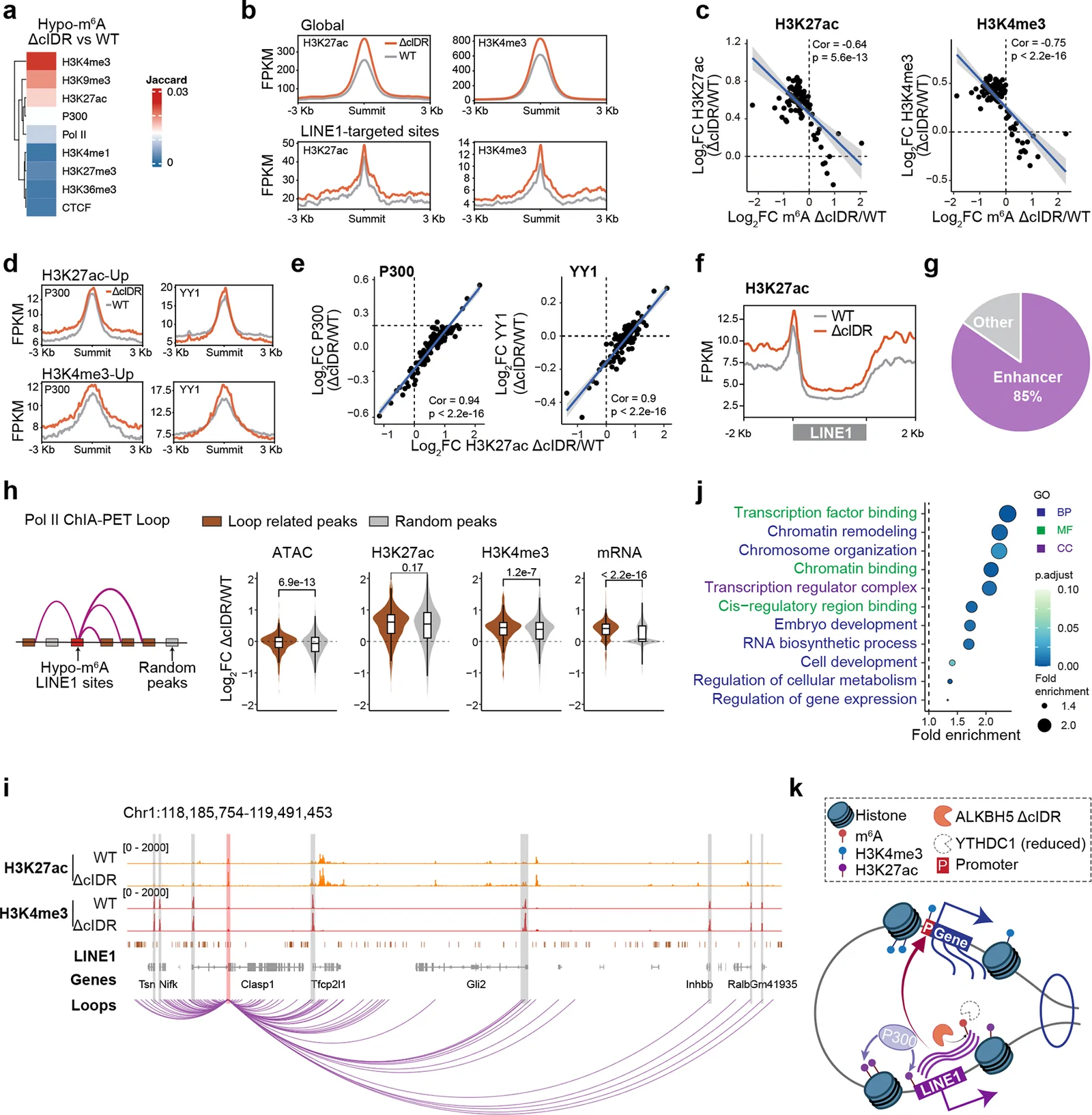
m^6^A-hypomethylated LINE1 acts as an enhancer-like element to modulate chromatin state in mESCs. **a**, Heatmap showing the Jaccard overlap between hypomethylated m^6^A peaks upon *Alkbh5*-ΔcIDR and ChIP-seq peaks of chromatin modifiers and histone modifications from GSE29218. **b**, Profiles of H3K27ac and H3K4me3 in WT and *Alkbh5*-ΔcIDR mESCs across genome-wide and at LINE1 RNA-targeted genomic sites, which were identified using LINE1 CHIRP-seq^28^. **c**, Correlation between changes in m^6^A levels and H3K27ac or H3K4me3 levels upon *Alkbh5*-ΔcIDR. **d**, Profiles of YY1 and P300 binding in WT and *Alkbh5*-ΔcIDR mESCs at upregulated H3K27ac and H3K4me3 regions upon *Alkbh5*-ΔcIDR. **e**, Correlation between changes in H3K27ac levels and P300 or YY1 binding upon *Alkbh5*-ΔcIDR. **f,** Aggregation plots showing H3K27ac levels across m^6^A-marked LINE1 elements in WT and *Alkbh5*-ΔcIDR mESCs. **g**, Proportion of loop-related m^6^A-hypomethylated LINE1 regions located within enhancers in *Alkbh5*-ΔcIDR mESCs. **h,** Left: schematic of “loop-related peaks” and “random peaks”. Peaks identified in ATAC-seq, H3K27ac and H3K4me3 CUT&Tag, and transcriptome data that overlapped with “m^6^A-hypomethylated LINE1-related loops” anchors were designated as ‘loop-related peaks’, while randomly selected non-interacting regions served as ‘random peaks’ for comparison. Right: violin plots showing fold changes in ATAC-seq, H3K27ac and H3K4me3 occupancy, and mRNA levels at loop-related peaks upon *Alkbh5*-ΔcIDR, two-tailed Mann–Whitney *U*-tests, n = 3 biological replicates. Box plots within violins show the median (centre line), IQR (box limits), and whiskers extending to 1.5× IQR. **i,** IGV visualization of histone modification changes at m^6^A-hypomethylated LINE1-related enhancer-promoter loops. m^6^A-hypomethylated LINE1 regions are highlighted in red, and promoter regions are highlighted in gray. **j**, Geno Ontology (GO) analysis of genes upregulated through m^6^A-hypomethylated LINE1-related loops in *Alkbh5*-ΔcIDR mESCs. BP, Biological Process; MF, Molecular Function; CC, Cellular Component **k**, Schematic model illustrating how ALKBH5-ΔcIDR-mediated m^6^A-hypomethylation of intergenic LINE1 elements modulates chromatin states. For (**c, e**), Pearson’s correlation test.

**Fig. 4:**
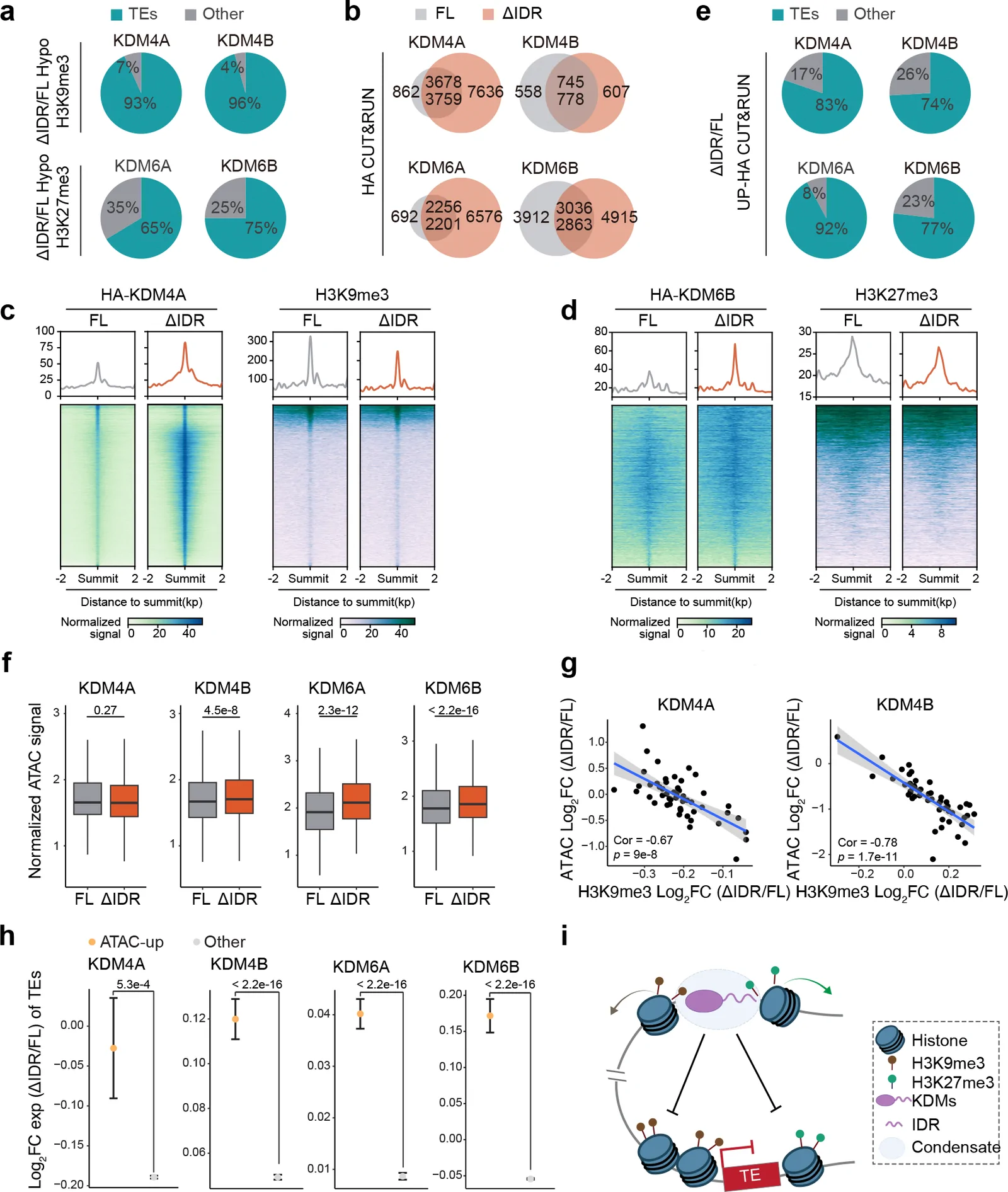
Deletion of IDRs in gene-activating histone demethylases leads to aberrant genomic targeting, chromatin opening and transcriptional activation of TEs. **a**, Proportion of downregulated H3K9me3 (for KDM4A/4B) and H3K27me3 (for KDM6A/6B) peaks that overlap with TEs, relative to the total number of downregulated peaks. Downregulated peaks are defined as those with reduced H3K9me3 (KDM4A/4B) or H3K27me3 (KDM6A/6B) in mESCs expressing ΔIDR variants compared to full-length (FL) variants. **b**, Overlap of genomic binding peaks identified by HA-tag CUT&RUN for HA-tagged FL and ΔIDR variants of KDM4A/4B and KDM6A/6B in mESCs. **c, d**, HA-tag CUT&RUN (left) and histone modification (right) signals in mESCs expressing FL or ΔIDR variants of KDM4A (**c**, H3K9me3) or KDM6B (**d**, H3K27me3). **e**, Proportion of increased HA-tag CUT&RUN peaks for KDM4A/4B and KDM6A/6B that overlap with TEs, relative to the total number of increased peaks. These increased peaks represent regions with higher HA-tag CUT&RUN signals in mESCs expressing ΔIDR versus FL variants. **f**, Relative ATAC-seq signals in mESCs ectopically expressing FL or ΔIDR variants of KDM4A/4B and KDM6A/6B. Box plots show the median (centre line), IQR (box limits), and whiskers extending to 1.5× IQR. **g**, Correlation between changes in chromatin accessibility (ATAC-seq) and H3K9me3 levels in mESCs expressing ΔIDR versus FL variants of KDM4A (left) or KDM4B (right). Pearson’s correlation test. **h**, Expression changes of repeat RNAs in ATAC-up versus other regions in mESCs expressing ΔIDR versus FL variants of KDM4A/4B or KDM6A/6B. ATAC-up regions are defined as regions showing increased ATAC-seq signals in mESCs expressing ΔIDR versus FL variants. Points represent medians; error bars indicate SEM. **i**, Schematic model showing the role of IDR within KDM4A/4B and KDM6A/6B in restricting demethylation on TEs, thereby preserving a repressive chromatin state. For (**f, h**), two-tailed Mann–Whitney *U*-tests, n = 3 biological replicates.

**Fig. 5:**
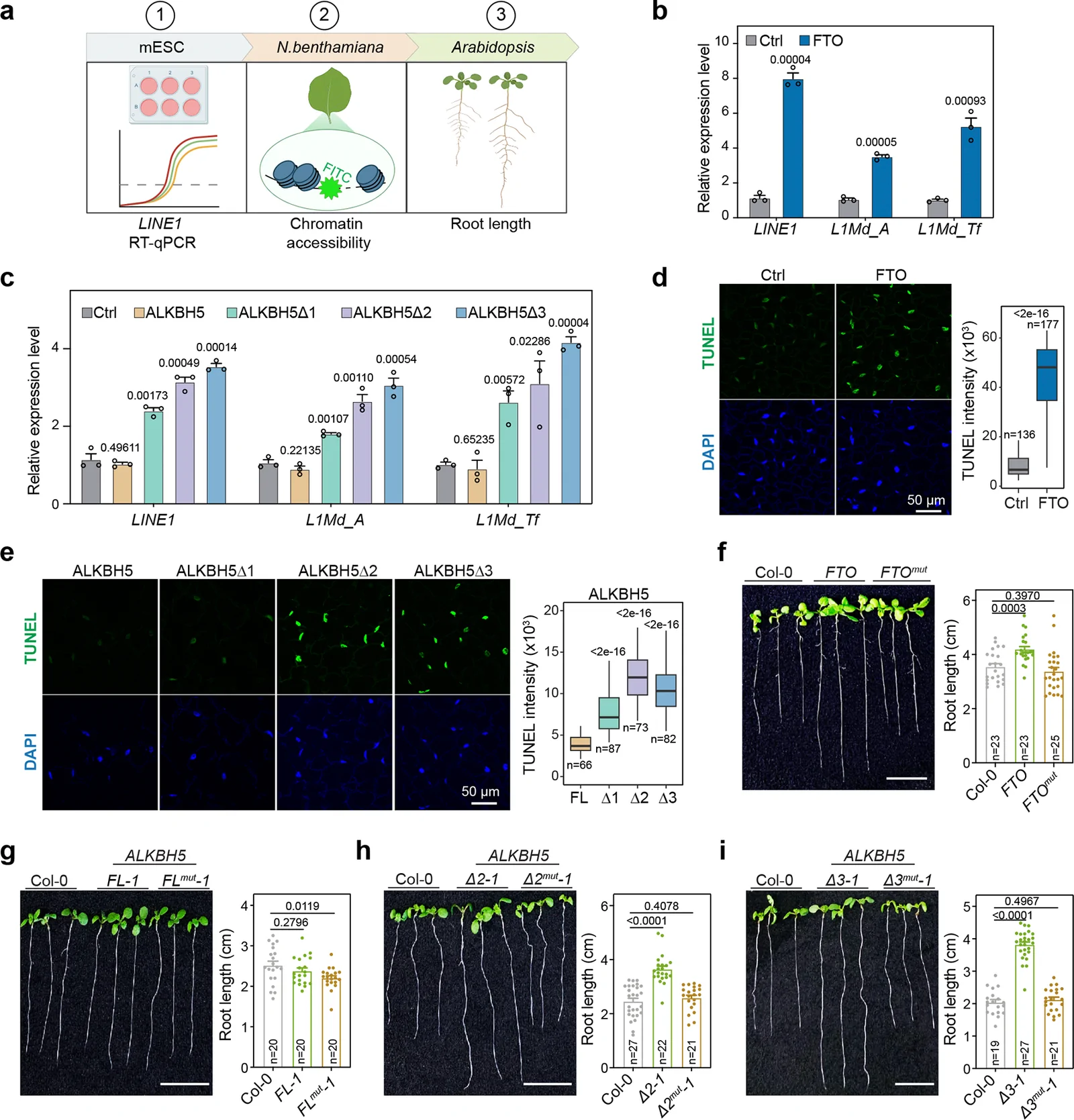
A systematic workflow for engineering ALKBH5 and its plant homologs for carRNA m^6^A demethylation. **a**, Schematic overview of the workflow for engineering ALKBH5 homologs. **b, c,** RT-qPCR analysis of whole-cell total LINE1 RNA and young LINE1 subfamilies RNAs in mESCs ectopically expressing human FTO (**b**) or human ALKBH5 variants (**c**). mESCs transfected with an empty vector served as controls (Ctrl). **d, e**, DNase I–treated TUNEL assays showing increased global chromatin accessibility in tobacco leaves transiently overexpressing human FTO (**d**) or IDR-deleted human ALKBH5 variants (**e**). Representative images are shown from three independent experiments. Scale bars, 50 μm. Boxplots represent relative TUNEL fluorescence intensity. **f-i**, Root growth phenotypes of 10-day-old *Arabidopsis* transgenic lines expressing human FTO (**f**) or human ALKBH5 variants (**g-i**). Representative images are shown on the left, and primary root length measurements are shown on the right. Scale bars, 1 cm. *FTO*^*mut*^ indicates R316Q/R322Q mutation. *FL*^*mut*^, *Δ2*^*mut*^ and *Δ3*^*mut*^ indicate ALKBH5 variants with H204A mutation. For (**c, e, g-i**), truncation variants of ALKBH5 correspond to specific residue deletions, as detailed in Extended Data Fig. 6a. Data are shown as means ± SEM (**b-c**, **f-i**) and for **b-i**, *p* values were calculated using unpaired two-tailed t tests. For **b-c**, n = 3 biological replicates. For **d-e**, box plots show the median (centre line), IQR (box limits), and whiskers extending to 1.5× IQR. Panel **a** was created using Biorender.com.

**Fig. 6:**
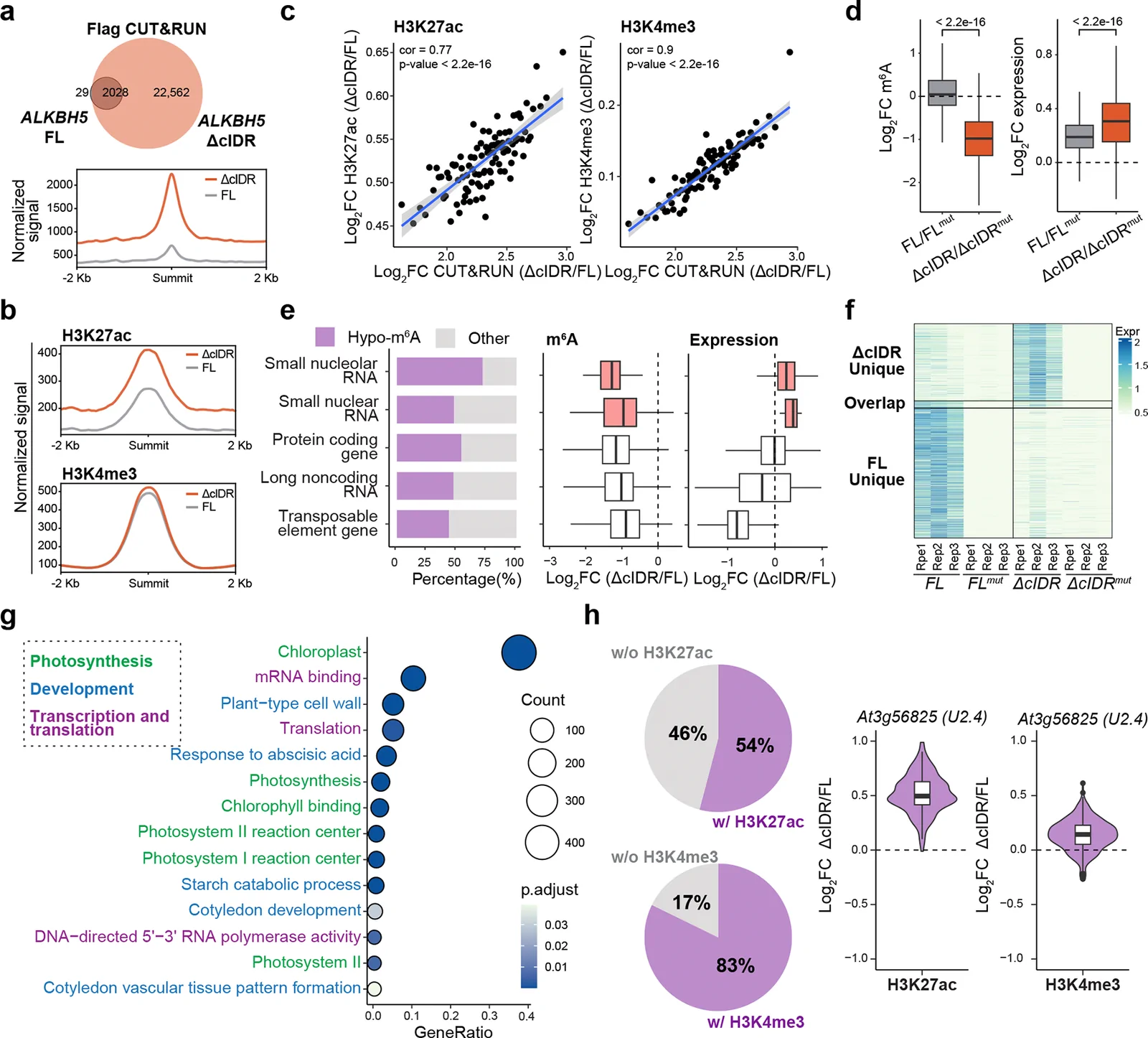
IDR-deleted ALKBH5 mediates caRNA m^6^A demethylation and chromatin activation in *Arabidopsis.* **a**, Top: Venn diagram showing the overlap of Flag CUT&RUN peaks between Flag-tagged ALKBH5 and ALKBH5-ΔcIDR in *Arabidopsis*. Bottom: Genome-wide profiles of normalized Flag CUT&RUN signals for ALKBH5 and ALKBH5-ΔcIDR. **b**, Genome-wide profiles of normalized H3K27ac (top) and H3K4me3 (bottom) signals in *ALKBH5*- and *ALKBH5*-ΔcIDR-*Arabidopsis*. **c**, Scatter plots showing the positive correlations between fold changes in Flag CUT&RUN signals and H3K27ac (left) or H3K4me3 (right) levels in *ALKBH5-*ΔcIDR- versus *ALKBH5*-*Arabidopsis*. Pearson’s correlation test was used. **d**, Fold changes in m^6^A levels (left) and expression levels of m^6^A-marked caRNAs (right) in *ALKBH5*- and *ALKBH5*-ΔcIDR-*Arabidopsis* relative to catalytically inactive mutant lines, two-tailed Mann–Whitney *U*-tests. **e**, Comparative analysis of m^6^A levels and caRNAs expression between *ALKBH5*-ΔcIDR- and *ALKBH5*-*Arabidopsis*. Left: percentage of hypomethylated m^6^A peaks across RNA categories in *ALKBH5*-ΔcIDR-*Arabidopsis* relative to *ALKBH5*-*Arabidopsis*. Middle: fold changes in m^6^A levels across RNA categories in *ALKBH5*-ΔcIDR- versus *ALKBH5*-*Arabidopsis*. Right: fold changes in expression levels across RNA categories in *ALKBH5*-ΔcIDR- versus *ALKBH5*-*Arabidopsis*. **f**, Heatmap of gene expression changes in *ALKBH5*- and *ALKBH5*-ΔcIDR-*Arabidopsis* compared to catalytically inactive mutant lines. **g**, GO enrichment analysis of upregulated genes in *ALKBH5*-ΔcIDR-*Arabidopsis* relative to *ALKBH5*-*Arabidopsis*. **h**, Chromatin states analysis of At*3g56825* (*U2.4*) interacted sites in *ALKBH5*- and *ALKBH5*-ΔcIDR-*Arabidopsis*. Left (pie chart): proportion of At*3g56825* interacted sites marked with H3K27ac or H3K4me3. Right (violin plot): fold changes of H3K27ac or H3K4me3 signals on At*3g56825* interacted sites in *ALKBH5-*ΔcIDR- versus *ALKBH5*-*Arabidopsis*. For **a-f**, FL represents full-length. For (**d-e, h**), box plots show the median (centre line), IQR (box limits), and whiskers extending to 1.5× IQR. For (**c-e, h**), n = 3 biological replicates.

**Fig. 7:**
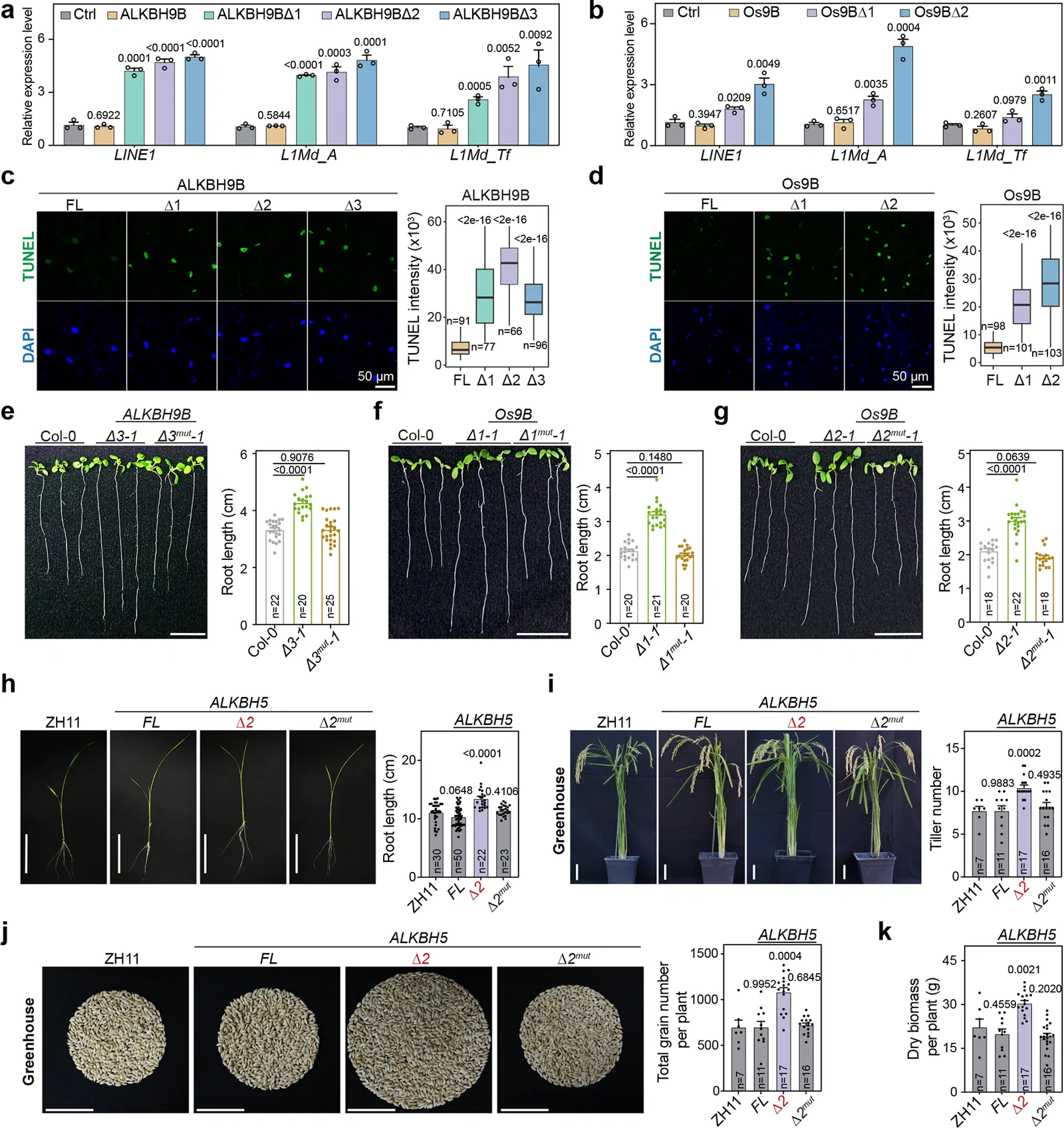
Engineering *Arabidopsis* and rice ALKBH5 orthologs to promote plant growth. **a, b**, RT-qPCR analysis of whole-cell total LINE1 RNA and young LINE1 subfamilies RNAs in mESCs ectopically expressing *Arabidopsis* ALKBH9B variants (**a**) or rice Os9B variants (**b**). mESCs transfected with an empty vector served as controls (Ctrl). **c, d**, DNase I–treated TUNEL assays showing increased global chromatin accessibility in tobacco leaves transiently overexpressing IDR-deleted variants of *Arabidopsis* ALKBH9B (**c**) or rice Os9B (**d**). Scale bars, 50 μm. **e-g**, Root growth phenotypes of 10-day-old *Arabidopsis* transgenic lines expressing *Arabidopsis* ALKBH9B variants (**e**) or rice Os9B variants (**f-g**). Representative images (left) and primary root length measurements (right) are shown. Scale bars, 1 cm. *ALKBH9BΔ3*^mut^ indicates the variant with H335A/D337A mutation; *Os9BΔ1*^mut^ and *Os9BΔ2*^mut^ indicate variants with H324A/D326A mutation. For **a-g**, details of *Arabidopsis* ALKBH9B and rice Os9B variants are provided in Extended Data Fig. 8d. **h**, Root length of 15-day-old WT (ZH11) and transgenic rice seedlings expressing human ALKBH5 variants grown under hydroponic culture conditions. **i-k**, Tiller number (**i**), total grain number (**j**), and dry biomass (**k**) per plant of WT (ZH11) and transgenic rice lines expressing human ALKBH5 variants grown in the greenhouse. For **h-j**, representative images are shown. Scale bars, 10 cm (**h-i**) and 4.5 cm (**j**). For (**a-b**, **e-k**), Data are shown as means ± SEM. For **a-k**, statistical significance was assessed using unpaired two-tailed t tests against the Ctrl (**a-b**), FL (**c-d**), Col-0 (**e-g**) or ZH11 group (**h-k**). For **a-b**, n = 3 biological replicates. For **c-d**, box plots show the median (centre line), IQR (box limits), and whiskers extending to 1.5×IQR. For **h-k**, *ALKBH5Δ2*^*mut*^ indicates H204A mutation; *ALKBH5*Δ2 refers to deletion of residues 298–394. For (**c-d**, **h-k**), FL represents full-length.
